# Refinement of SARS-CoV-2 envelope protein structure in a native-like environment by molecular dynamics simulations

**DOI:** 10.3389/fmolb.2022.1027223

**Published:** 2022-10-10

**Authors:** Rui Yang, Sijin Wu, Shen Wang, Grace Rubino, Jonathan D. Nickels, Xiaolin Cheng

**Affiliations:** ^1^ Division of Medicinal Chemistry and Pharmacognosy, College of Pharmacy, The Ohio State University, Columbus, OH, United States; ^2^ Department of Chemical and Biomolecular Engineering, College of Engineering, The Ohio State University, Columbus, OH, United States; ^3^ Department of Chemical and Environmental Engineering, The University of Cincinnati, Cincinnati, OH, United States; ^4^ Translational Data Analytics Institute (TDAI), The Ohio State University, Columbus, OH, United States

**Keywords:** COVID-19, coronavirus, envelope (E) protein, SARS-CoV-2, E protein inhibitors

## Abstract

COVID-19 has become an unprecedented threat to human health. The SARS-CoV-2 envelope (E) protein plays a critical role in the viral maturation process and pathogenesis. Despite intensive investigation, its structure in physiological conditions remains mysterious: no high-resolution full-length structure is available and only an NMR structure of the transmembrane (TM) region has been determined. Here, we present a refined E protein structure, using molecular dynamics (MD) simulations to investigate its structure and dynamics in a 1-palmitoyl-2-oleoyl-sn-glycero-3-phosphocholine (POPC) bilayer system. Our initial homology model based upon the SARS-CoV E protein structure is shown to be unstable in the lipid bilayer, and the H3 helices tend to move away from the membrane center to the membrane-water interface. A more stable model was developed by replacing all H3 helices with the fully equilibrated H3 structure sampled in the MD simulations. This refined model exhibited more favorable contacts with lipids and water than the original homology model and induced local membrane curvature, decreasing local lipid order. Interestingly, the pore radius profiles showed that the channel in both homology and refined models remained in a closed state throughout the simulations. We also demonstrated the utility of this structure to develop anti-SARS-CoV-2 drugs by docking a library of FDA-approved, investigational, and experimental drugs to the refined E protein structure, identifying 20 potential channel blockers. This highlights the power of MD simulations to refine low-resolution structures of membrane proteins in a native-like membrane environment, shedding light on the structural features of the E protein and providing a platform for the development of novel antiviral treatments.

## Introduction

Over 2 years have passed since the outbreak of the severe acute respiratory syndrome coronavirus 2 (SARS-CoV-2), which is also referred to as the novel coronavirus disease (COVID-19). As of January 2022, there have been over 300 million confirmed cases of COVID-19 including over five million deaths. Thanks to the strict social distancing in many countries and the rollout of vaccines from Pfizer/BioNtech, Moderna, and Johnson & Johnson, etc., the spread of the virus has been tremendously slowed down. However, the emergence of several SARS-CoV-2 variants, such as the Delta and Omicron variants has caused renewed COVID outbreaks, increasing acute concerns and reminding the world to maintain efforts to find new and better therapeutics and antiviral agents.

The genome of SARS-CoV-2 encodes three membrane proteins: the spike (S) protein, the membrane (M) protein, and the envelope (E) protein. S protein binds to the receptor on host cell surface and mediates viral entry (Zhou et al., 2020; Jackson et al., 2022). M protein plays a critical role in virus assembly and budding processes (Weiss and Navas-Martin, 2005; Plescia et al., 2021). E protein is composed of 75 amino acids and interacts with M protein, which is important for viral formation and release (Lim and Liu, 2001; Hsieh et al., 2008). The E protein is an integral membrane protein. It forms a pH-sensitive pentameric cation channel on the ERGIC/Golgi membrane, which is permeable to Ca^2+^, Na^+^, and K^+^ (Verdiá-Báguena et al., 2012; Nieto-Torres et al., 2015; Cabrera-Garcia et al., 2021; Verdiá-Báguena et al., 2021; Xia et al., 2021). Deletion of the E protein in recombinant coronaviruses resulted in significantly reduced viral titers and viral maturation, while mutations of the E protein affected post-translational modification and impaired ion conductivity, leading to reduced viral formation and pathogenicity (DeDiego et al., 2007; Boscarino et al., 2008; Lopez et al., 2008; Verdiá-Báguena et al., 2012; DeDiego et al., 2014; Castaño-Rodriguez et al., 2018). The E protein, together with the M protein, modulates the N-glycosylation of the S protein and is essential for the optimal assembly of viral particles (Siu et al., 2008; Boson et al., 2021). The E protein alone was found to cause cell death *in vitro* and trigger strong immune responses *in vivo* (Xia et al., 2021). A recent study demonstrated that the SARS-CoV-2 E protein stimulates the production of an inflammatory chemokine by binding and activating the TLR2 signaling cascade (Planès et al., 2022). Thus, it is hypothesized that inhibition of the E protein’s ion channel activity could disrupt viral assembly, decrease viral maturation, and eventually reduce viral infection. Several studies have identified potential E protein viroporin blockers, some of which exhibited protective effects *in vitro* against SARS-CoV/SARS-CoV-2, such as hexamethylene amiloride, amantadine, glicazide, and tretinoin, etc (Pervushin et al., 2009; Dey et al., 2020; Mandala et al., 2020; Singh Tomar and Arkin, 2020; Das et al., 2021; Park et al., 2021; Xia et al., 2021). Compared to the heavily studied S protein which has exhibited rapid mutation that often correlates with enhanced infection and death rates, the E protein appears to be more stable and conserved among detected variants. The E protein is also less thoroughly investigated as a therapeutic target for vaccine and drug development against coronavirus (Alam et al., 2020; Tilocca et al., 2020; Das and Roy, 2021; Troyano-Hernáez et al., 2021). The stability of the E protein may point to it being a better long-term target for therapeutic intervention against SARS-CoV-2.

**FIGURE 1:**
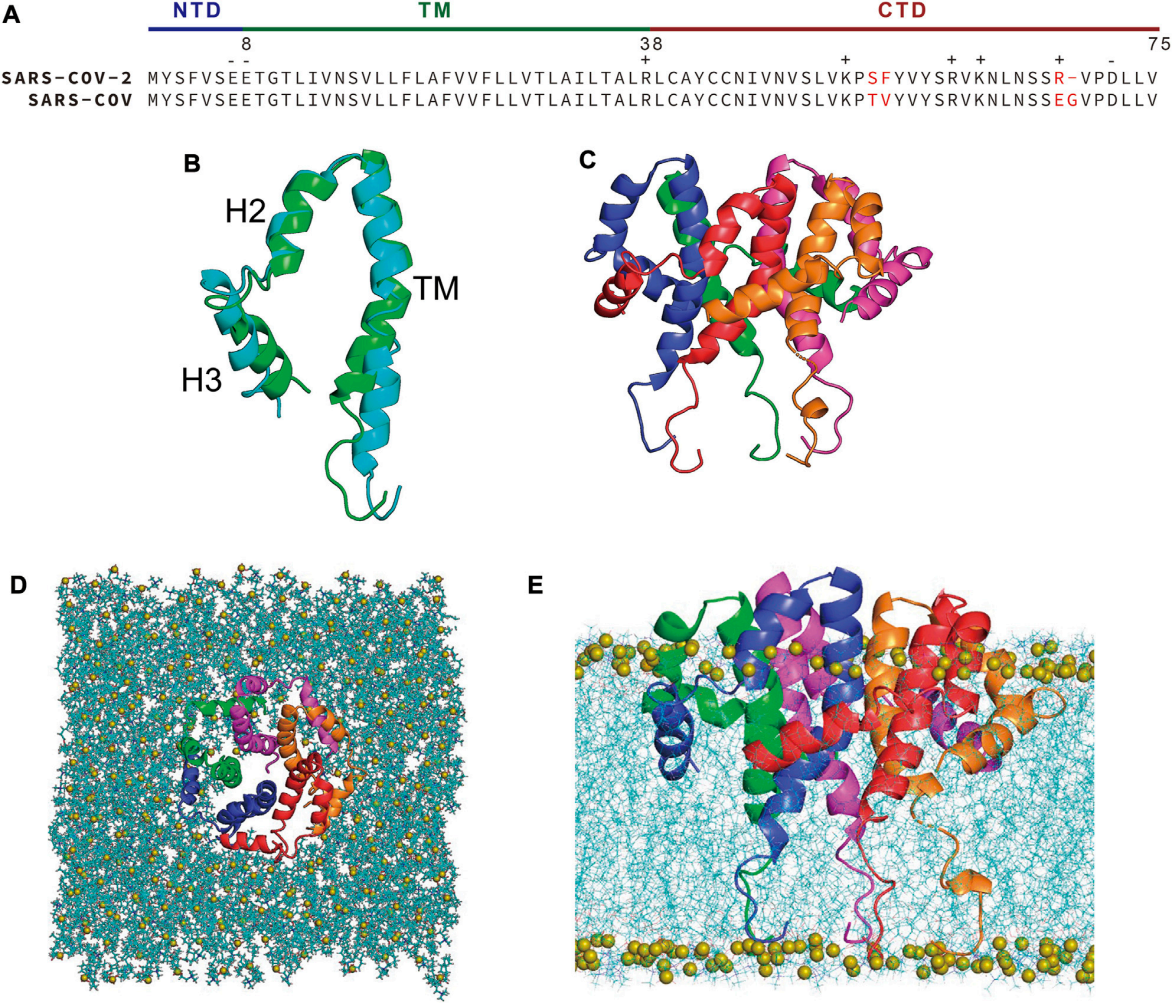
**(A)** Sequence alignment of SARS-CoV-2 and SARS-CoV E protein. Non-conserved residues are highlighted in red. **(B)** Superposition of the homology model of SARS-CoV-2 E protein (green) and the NMR structure (PDB code: 5X29) of SARS-CoV E protein (cyan). **(C)** Assembled pentameric model of SARS-CoV-2 E protein. **(D)** Top view and **(E)** side view of the SARS-CoV-2 E protein embedded in a POPC bilayer. Chain A-E are colored with red, blue, green, magenta and orange, respectively. Lipids are shown as blue lines, with the phosphorus atoms of the lipid shown as olive spheres.

The structure of the E protein can be three regions: the N-terminal domain (NTD), the transmembrane domain (TMD), and the C-terminal domain (CTD) (Figure 1A) (Schoeman and Fielding, 2019). The TM regions oligomerize into a homopentameric channel (Pervushin et al., 2009; Surya et al., 2018; Mandala et al., 2020). Mutations in the hydrophobic TMD, such as N15A and V25F, led to the abrogation of ion conductance (Verdiá-Báguena et al., 2012). The CTD, facing the cytoplasm, contains a PDZ-binding motif (final four amino acid residues DLLV), which can potentially bind to over 400 host cellular proteins that carry a PDZ domain, like PALS1 or ZO1 (Teoh et al., 2010; Münz et al., 2012; Castaño-Rodriguez et al., 2018; Chai et al., 2021; Shepley-McTaggart et al., 2021). Although the SARS-CoV-2 E protein has been intensively studied since the outbreak of COVID-19, its structure remained largely unknown until a high-resolution solid-state NMR structure of its TM region was reported in late 2020 (PDB code: 7K3G) (Mandala et al., 2020). A full-length (PDB code: 2MM4) and a near full-length (residues 8-65) (PDB code: 5X29) solution NMR structures of its close analog, the SARS-CoV E protein, were determined in sodium dodecyl sulfate (SDS) and 1-myristoyl-2-hydroxy-sn-glycero-3-phospho-(1′-rac-glycerol) (LMPG) micelles, respectively, which could serve as good templates for homology modeling of the SARS-CoV-2 E protein (Li et al., 2014; Surya et al., 2018).

Here, we built a homology model of SARS-CoV-2 E protein and applied microsecond-long all-atom molecular dynamics (AA-MD) simulations to study the structural and dynamic properties of the SARS-CoV-2 E protein in a model 1-palmitoyl-2-oleoyl-sn-glycero-3-phosphocholine (POPC) bilayer. Our study indicated that MD simulations can be employed to refine NMR structures determined from non-native environments. The refined E protein structure allowed us to explore the interplay between the E protein and the membrane in which it natively exists. The refined structure also provided a structural basis to start identifying potential channel blockers by molecular docking of a library of FDA-approved, investigational and experimental drugs against the E protein.

## Results and discussion

### SARS-CoV-2 E protein was constructed through homology modeling and assembled into a lipid bilayer

The full-length structure of SARS-CoV-2 E protein remains ambiguously defined despite numerous investigations. A partial solid-state NMR structure of the protein, the TM domain, was determined by Hong’s group (PDB code: 7K3G) (Mandala et al., 2020), while Surya et al. reported a solution NMR structure of the SARS-CoV E protein in LMPG micelles (PDB code: 5X29), which covered residues 8-65 (Surya et al., 2018). Since the overall and TMD of the SARS-CoV and SARS-CoV-2 E proteins share 94.7% and 100% sequence identity, respectively (Figure 1A), we built a homology model of the near full-length SARS-CoV-2 E protein (residues 8-65) with MODELLER (Šali and Blundell, 1993) using the 5X29 structure as the template (Figure 1B). The homology model has the same topology as the template: a transmembrane α-helix (residue 8-38) followed by two short α-helices (H2 and H3) connected by a flexible linker in the CTD (residue 39-65). The stereochemical quality of the homology model was validated by SAVES v6.0 developed by DOE lab at UCLA (https://saves.mbi.ucla.edu/) and Molprobity developed by Williams et al. at Duke University (http://molprobity.biochem.duke.edu/) (Williams et al., 2018) and the results were summarized in Supplementary Table S1. All residues in our homology model fall in the allowed regions and the ERRAT overall quality factor is 88 with a 3.01 clash score, indicating that the homology model has an acceptable quality. Interestingly, the two NMR structures have a relatively low ERRAT overall quality factor and 3.6% of 7K3G’s residues even fall in the disallowed regions. The other three homology models all have comparable or slightly better qualities than our homology model. Thus, we think that our homology model can be reliably used as the starting structure for MD simulations.

The homology model was assembled into a pentameric complex (Figure 1C) and subsequently inserted into a 1-palmitoyl-2-oleoyl-sn-glycero-3-phosphocholine (POPC) bilayer using the CHARMM GUI webserver (Figures 1D,E) (Jo et al., 2008; Wu et al., 2014). However, visual inspection of the assembled system suggests that some polar and charged residues in the CTDs of the E protein are buried in the lipid bilayer and thus have unfavorable interactions with the hydrophobic lipid tails. This unphysical arrangement of the CTDs might result from the non-native condition in which the SARS-CoV E protein was determined - detergent micelles - instead of more native-like phospholipid bicelles or nanodiscs (Lau et al., 2009; Suk et al., 2012; Surya et al., 2013). Thus, we aimed to refine the homology model of SARS-CoV-2 E protein by all-atom molecular dynamics (AA-MD) simulations. Three separate simulations were performed for the E protein homology model embedded in a POPC bilayer at 303K for 2 µs each. An additional simulation of the same system was performed at 323 K for 1 µs in order to speed up the conformational sampling.

### SARS-CoV-2 E protein displays significant structural fluctuations in AA-MD simulations

**FIGURE 2:**
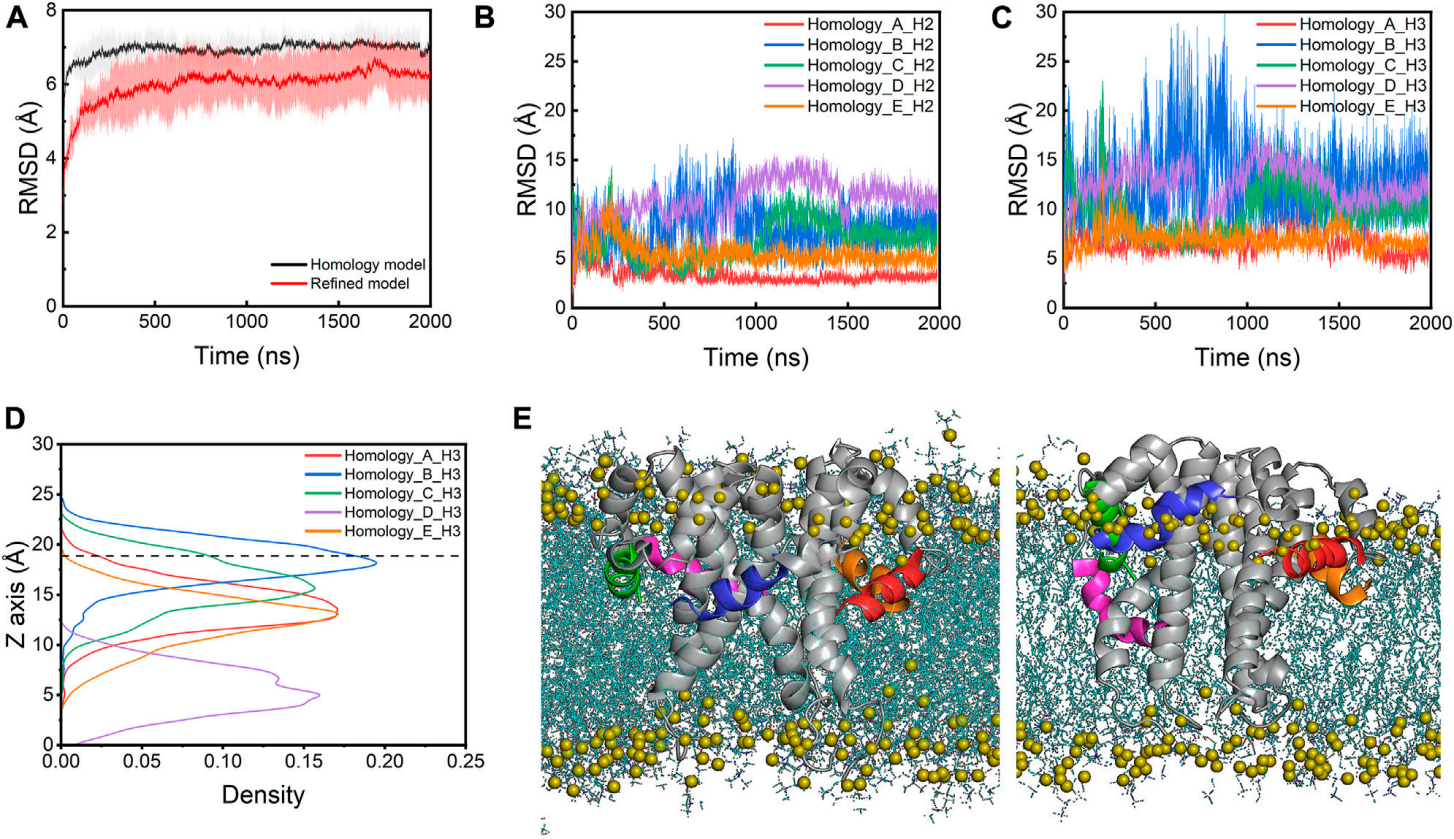
**(A)** RMSDs of the homology model and the refined model. The lines are the mean values averaged from the three parallel simulations. The shade area is the standard deviation. RMSDs of **(B)** the H2s and **(C)** the H3s of the homology model. **(D)** The density profile of the centers of mass (COMs) of H3s along the membrane normal in the simulations of the homology model. The average position of the phosphorus atoms on the upper leaflet is shown as a dashed line for reference. **(E)** The initial (left) and last frame (right) of the homology model in a POPC bilayer from the AA-MD simulations. H3 helices of chain A-E are colored with red, blue, green, magenta and orange, respectively. Lipids are shown as blue lines and the phosphorus atoms are shown as olive spheres.

During the simulations, the E protein (referred to as the homology model below) displays significant structural fluctuations as indicated by the large root mean square deviation (RMSD) values of the heavy atoms, quickly rising to ∼7 Å within the first 50 ns (Figure 2A). The CTDs contribute more to the structural fluctuations as indicated by the larger RMSDs (>5 Å) than the TMs (∼3Å) throughout the simulations (Supplementary Figures S1A, S1C). A similar fluctuation pattern is observed in the root mean square fluctuations (RMSFs) of the Cα atoms, where the RMSFs of CTDs are much higher than those of the TM regions (Supplementary Figure S2A). Within the CTDs, the H3 helices fluctuate more than the H2 helices (Figures 2B,C). During the simulations, all five H2s stay at the membrane-water interface, but the H3s of four monomers (chain A, B, C and E) move away from the hydrophobic core toward the lipid headgroups while only one H3 (chain D) does not display any significant upward or downward movement and remains at the lipid tailgroup level (Figures 2D,E). This movement is likely due to the incompatibility between the polar and charged residues in the CTDs and the hydrophobic lipid core, suggesting that the NMR structure determined in detergent micelles does not represent a functionally relevant conformation of the E protein in its native environment.

### A refined SARS-CoV-2 E protein model shows smaller structural fluctuations

**FIGURE 3:**
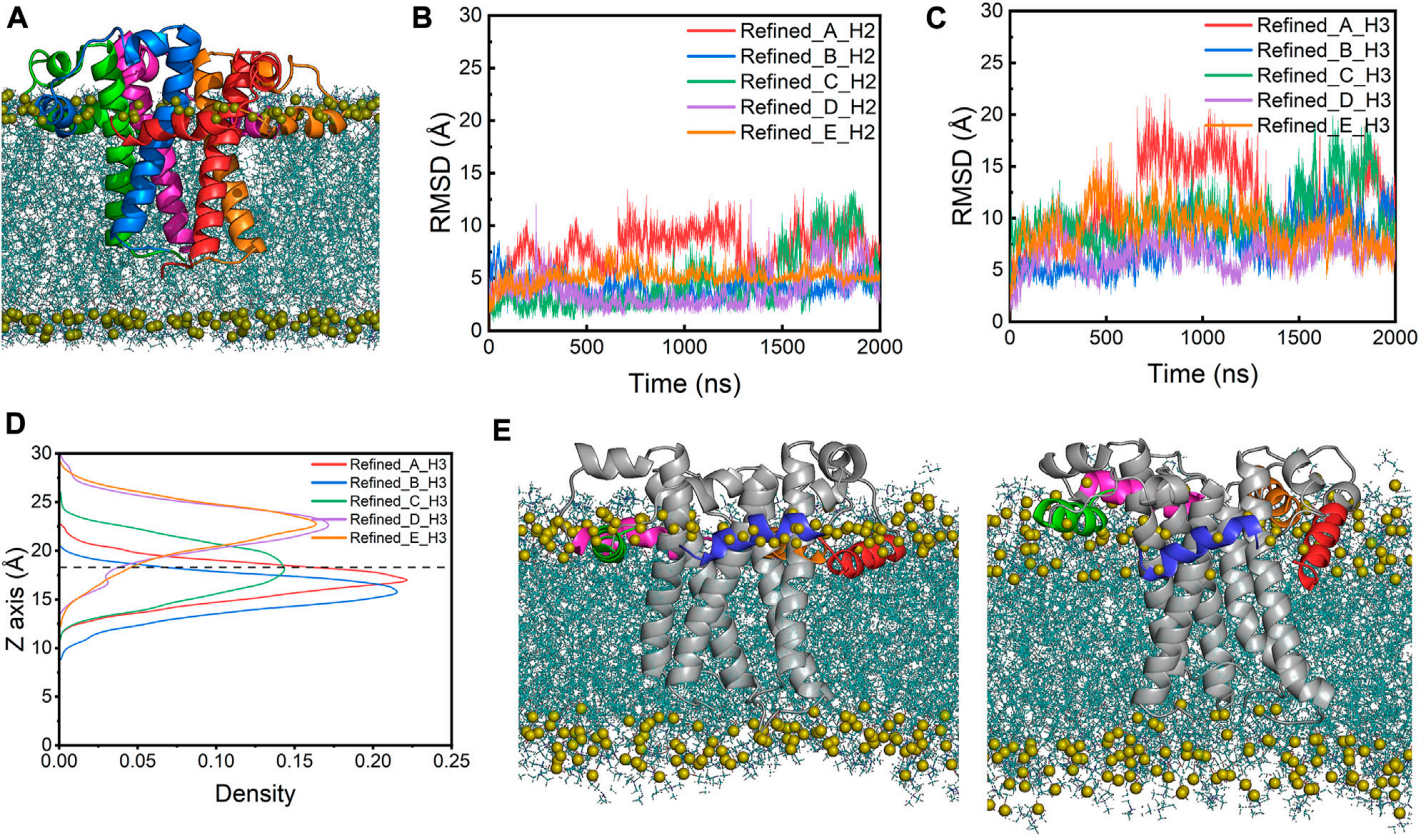
**(A)** The refined model assembled in a POPC bilayer (side view). RMSDs of **(B)** the H2s and **(C)** the H3s of the refined model. **(D)** The density profile of the H3s COMs along the membrane normal in the simulations of the refined model. The average position of the phosphorus atoms on the upper leaflet is shown as a dashed line for reference. **(E)** The initial (left) and last frame (right) of the refined model in a POPC bilayer from the AA-MD simulations. The representation and color scheme are the same as Figure 2E.

Simulation of the homology model of the SARS-CoV-2 E protein showed that the homology modeling approach did not result in a realistic conformation in the membrane environment. Thus, we built a new structural model of the SARS-CoV-2 E protein by taking a structural frame at ∼500 ns from the 323K simulation trajectory, in which the H3 of its chain C has completely moved to the membrane-water interface. We replicated this chain C model four times and assembled the resulting five protomers into a pentamer using the initial homology model as a template. The new pentameric model (referred to as the refined model below) was subsequently inserted into the POPC bilayer (Figure 3A). In the refined model, all H3s were located at the membrane-water interface, with H2s partially unfolded. Three MD simulations of the refined model were performed at 303K for 2 µs each. The overall RMSD values of the refined model are smaller than those of the homology model (Figure 2A). Particularly, compared to the original homology model, the refined model displays smaller RMSDs and RMSFs for both TMDs and CTDs (both H2s and H3s) (Figures 3B, 3C; Supplementary Figures S1B, S1D, S2B), indicating that the new model is more stable. This is likely due to the elimination of the unfavorable interactions between CTDs and lipid tails in the homology model. H2s stay in the water layer while H3s remain at the membrane-water interface for most of the time throughout the simulations (Figures 3D,E), only occasionally dissociating and re-associating from the interface area, indicated by the sudden increase of RMSD and the movement of the centers of mass (COMs) along the membrane normal (Figure 3C; Supplementary Figure S3B). Interestingly, the TM helices in the refined model are found to tilt slightly more than those in the homology model, which might also facilitate the H3s approaching the membrane headgroup region (Supplementary Figure S4).

**FIGURE 4:**
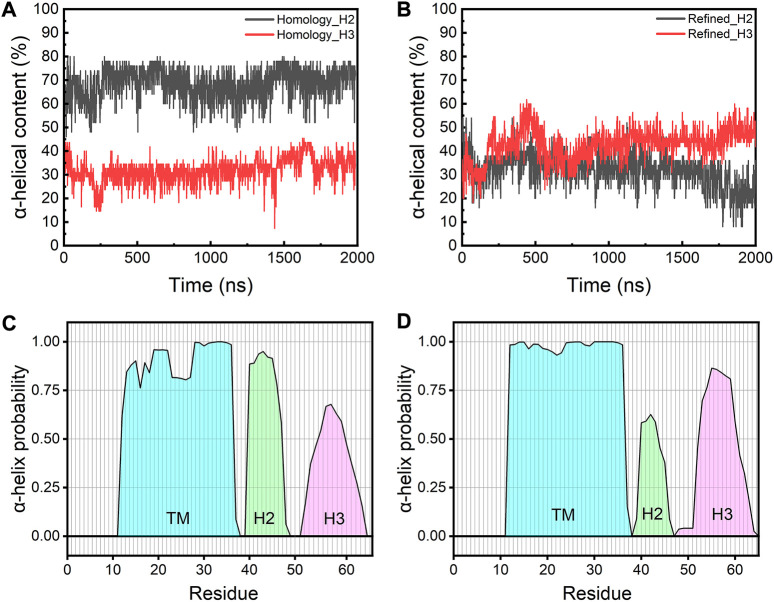
Time-evolution of α-helical content of H2s and H3s in **(A)** the homology and **(B)** the refined model. Average probability of the α-helical structure for each residue from simulations of **(C)** the homology model and **(D)** the refined model. Cyan: TM regions; green: H2 helices; magenta: H3 helices.

We next calculated the α-helical probability for each residue from the AA-MD simulations. The secondary structure content of the TM domains is largely maintained in both models during the simulations, while the TM regions in the refined model show a slightly higher α-helical content than those in the homology model (∼80% vs. ∼70%) (Supplementary Figure S5). In contrast, the H2 helices in the refined model are found to be less structured than those in the homology model. This is probably because all H2 helices in the refined model are fully immersed in the water layer, which caused them to be at fast unfolding/refolding equilibrium, while those helices in the homology model are only partially above the membrane surface and remain largely folded in an amphiphilic environment (Figures 4A,B). Unlike the H2 helices, the H3 helices in the refined model show a slight increase of the α-helix content when comparted to the homology model. This may be because these helices can make more favorable interactions with lipid headgroups in the refined model than in the homology model where the H3s are trapped in a hydrophobic environment and are thus energetically less stable (Figures 4C,D). These results are in good agreement with a recently published study where the authors demonstrated the secondary structure of the TM regions is mostly maintained while the H2s and H3s are more disordered throughout the atomistic MD simulations (Kuzmin et al., 2022).

### The refined model is stabilized by more H bonds and contacts with lipids/water

**FIGURE 5:**
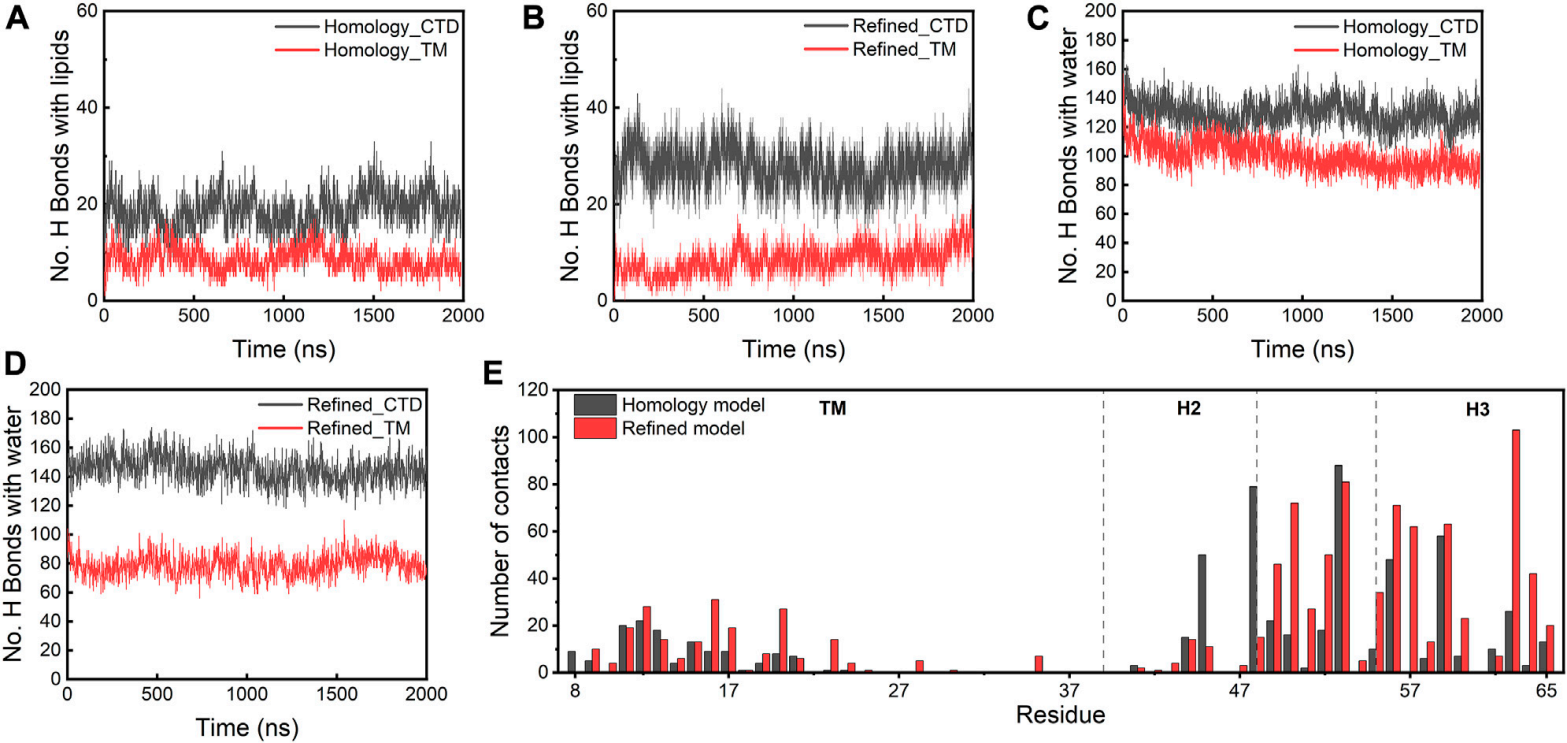
**(A–D)** H bonds formed between lipids/water and the homology and refined models. **(E)** Total number of contacts with lipids per residue (within a 4 Å cut-off) in both models.

To understand how the refined model is stabilized relative to the homology model, we examined the molecular interactions between the protein complex and lipid or water molecules in the local environment. In both models, the CTDs establish more H bonds with lipids and water than the TM regions (Figures 5A–D). The CTDs in the refined model form more H bonds (∼30) with lipids than in the homology model (∼20) because the CTDs in the refined model were in closer proximity to lipid headgroups. Figure 5E depicts the number of contacts with lipids for individual residues of the E protein, which confirms that the majority of residues involved in the protein-lipid interactions is located in the CTD regions and the H3s form more contacts with lipids in the refined model than in the homology model. Strikingly, the TM domains in the refined model form fewer H bonds with water than those in the homology model, while the number of H bonds between the TM regions and lipids is approximately equal for both models. Close inspection reveals a substantial amount of water molecules in the homology model moving into the lipid bilayer and staying in the vicinity of the protein. This appears to be due to the burial of the polar H3s in the membrane hydrophobic core which greatly destabilizes the lipid bilayer. In contrast, the lipid bilayer remains intact with no water molecules trapped inside the membrane throughout the simulations of the refined model (Supplementary Figure S6). These data show that the integrity of the membrane is dependent on the presence of the different protein models, substantiating that the refined model is likely a more physiologically-relevant conformation of the E protein in the membrane environment than the original homology model.

### E protein induces local membrane curvature

**FIGURE 6:**
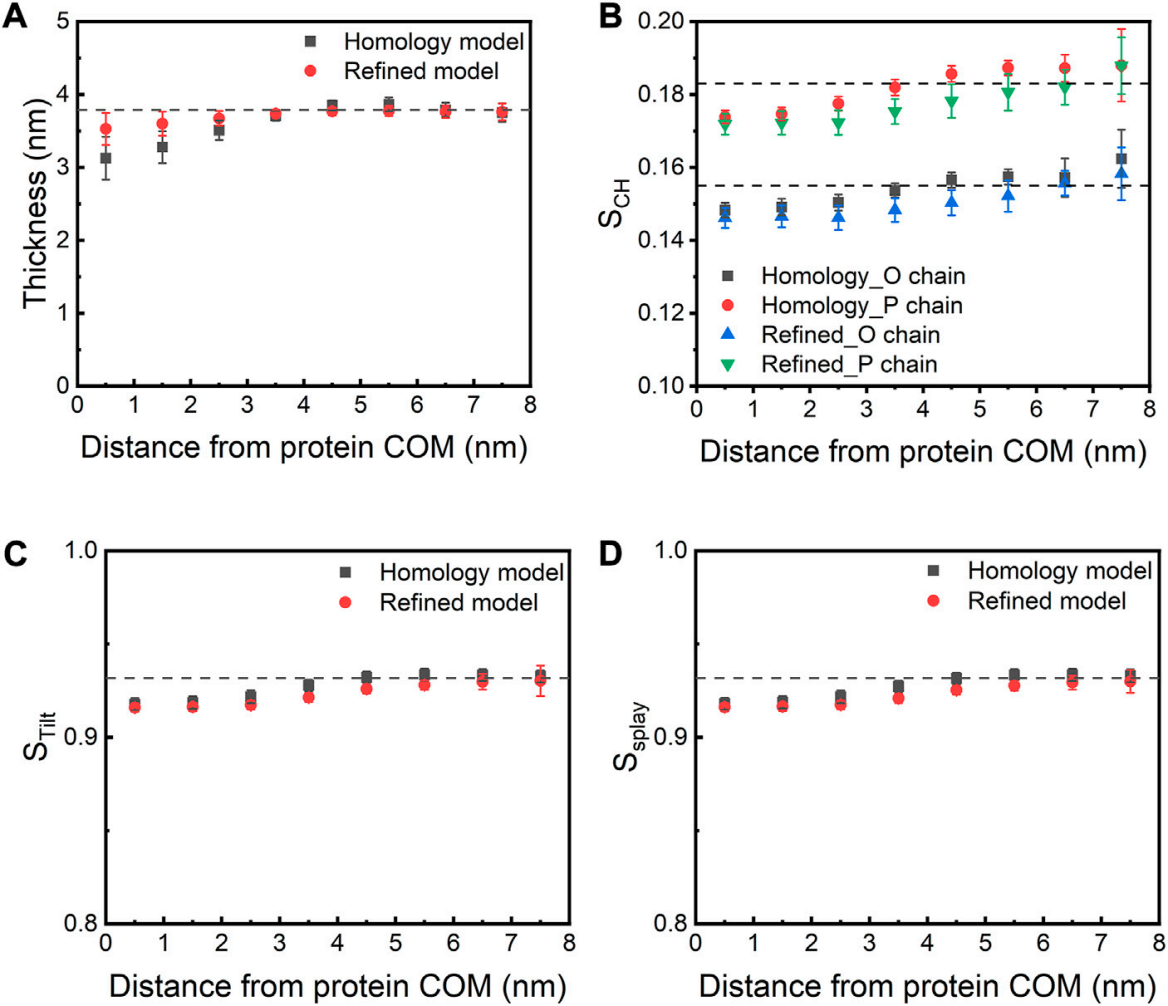
**(A)** Membrane thickness and **(B)** order parameter as a function of the distance from the COM of the E protein. The dashed lines indicate the corresponding values calculated from the AA-MD simulation (200 ns) of a pure POPC bilayer. **(C)** S_Tilt_ and **(D)** S_Splay_ as a function of the distance from the COM of the E protein. S_Tilt_ is calculated as the average tilt angle of a lipid with respect to the bilayer normal. S_Splay_ reflects the divergence of local lipids tilt.

We next investigated how the presence of the E protein affects various membrane properties. We first calculated the bilayer thickness as a function of the distance from the COM of the E protein (Figure 6A). Compared to a pure bilayer system, the bilayer thickness decreases within ∼3 nm of the protein COM in both models, while the presence of the protein seems to have little or no effects on the bilayer thickness between 4 and 6 nm from the protein COM (the largest radius of the protein is ∼2.5 nm), suggesting that the bilayer around the E protein bends toward the membrane core. It is noteworthy that, along with the movement of the E protein, especially H3s, this membrane bending motion also contributes to bringing the H3 helices close to the membrane-water interfacial region. These results are in agreement with Kuzmin et al.‘s work, where the authors found that the induction of membrane curvature by the E protein led to thinner membrane close to the TM regions but thicker around the CTDs (Kuzmin et al., 2022). Yet, another study by Collins et al. found that the E protein was not able to facilitate membrane curvature (Collins et al., 2021). These inconsistent results might arise from the different protein structural models (Heo and Feig’s predicted model (Heo and Feig, 2020) vs. our refined model generated from MD simulations) or the different membrane systems used in the studies (ERGIC mimic vs. pure POPC).

In parallel to bilayer thinning, a decrease in the order parameter (S_CH_) of lipid acyl chains is found in both models when compared to the pure POPC bilayer, especially for lipids within 1-4 nm of the protein. This indicates that the protein makes the surrounding lipids more flexible and less ordered (Figure 6B). We also observed that both models decrease the tilt (S_Tilt_) and splay (S_Splay_) parameters of surrounding lipids (Figures 6C,D). Since lipid tilt and splay are known to be correlated with membrane local rigidity (Watson et al., 2012; Khelashvili et al., 2013), these results suggest that the E protein can soften local lipid bilayer.

Taken together, our simulations show interplay between SARS-CoV-2 E protein and the membrane, which rearranges the position/orientation of the H3 helices relative to the membrane, eventually leading to more favorable accommodation of the protein in the membrane. This rearrangement is achieved through a mutual adaptation mechanism. The E protein relieves frustration due to the burial of its H3s in the membrane, through the movement of the H3 helices and the shift/tilting of the TM helices, while the membrane adapts to the protein by changing its thickness and lipid order. The E protein is known to play a critical role in the viral maturation and budding process of SARS-CoV-2 infection. However, the exact mechanism remains poorly understood. Our results reveal that the presence of the protein induces a local thinning and softening of the surrounding lipid bilayer, which in turn facilitates accommodating the TM helices comprising polar residues on both termini that need to be solvent-exposed. This window into the complexity of the E protein-membrane interaction provides an additional basis for understanding how the E protein contributes to the viral budding process.

### The viroporin channel remains in a closed state in both models

**FIGURE 7:**
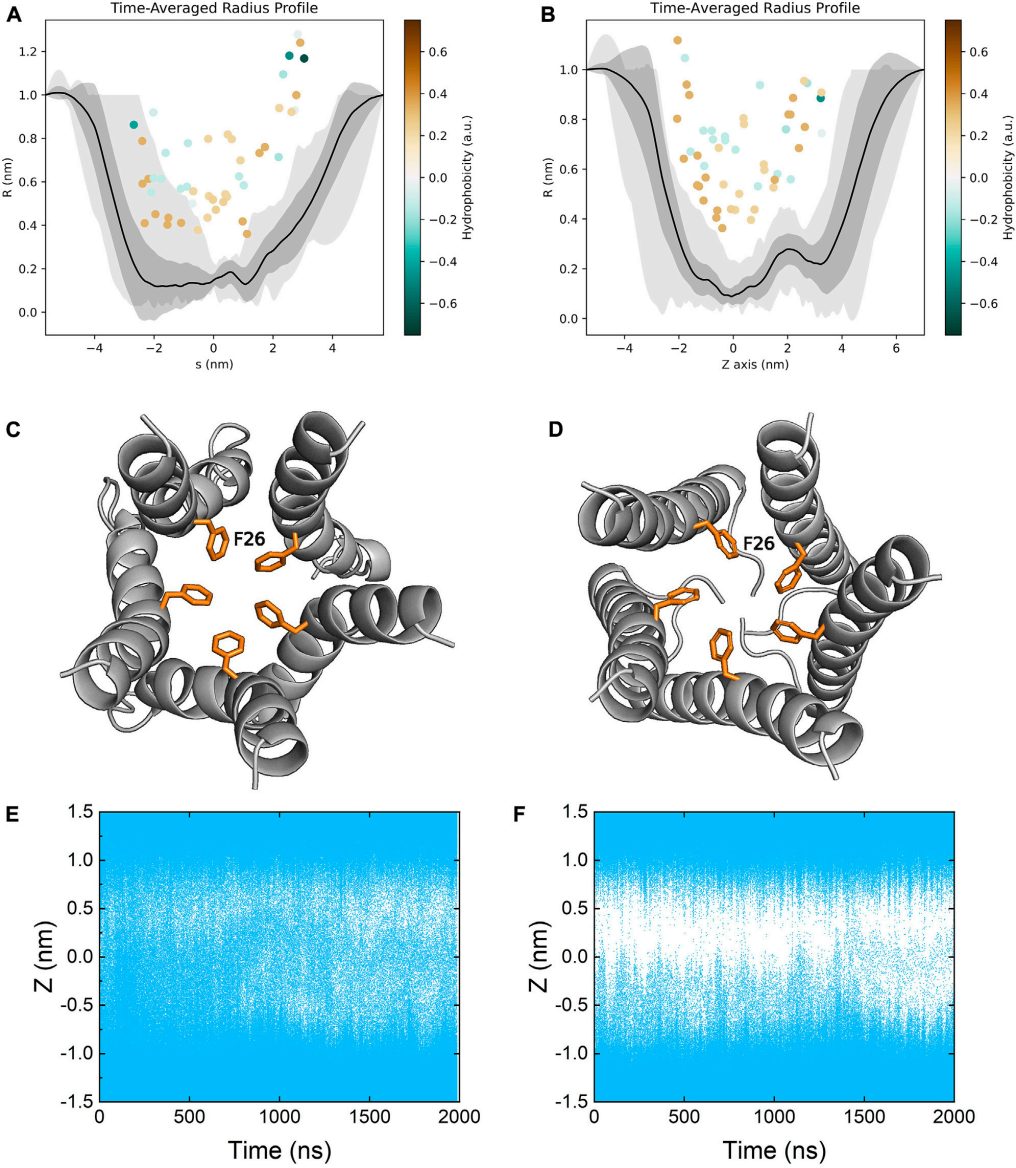
Time-averaged pore radius profiles of **(A)** the homology model and **(B)** the refined model. The grey shaded areas correspond to the one-sigma (dark gray) and two-sigma (light gray) confidence intervals. The pore lining residues (facing the channel more than 50% of the time) are indicated as dots colored by their hydrophobicity. Top view of the bottleneck residue PHE26 in the channel of **(C)** the homology model and **(D)** the refined model. Pore hydration profiles of **(E)** the homology model and **(F)** the refined model. Coordinates of water molecules along the *z* axis were plotted as a function of time. Each water molecule was represented by a blue dot.

The ion channel of the E protein is lined primarily with hydrophobic residues (e.g., LEU18, ALA22, PHE26, VAL29, ILE33 and LEU37) and two hydrophilic residues (i.e., GLU8 and ASN15). We calculated the channel radius profiles of both models (Figures 7A,B). Since the TM domains are relatively stable during the simulations, the pore radius profiles of both models are similar to each other. PHE26 is identified as the bottleneck (narrowest) residue in the middle of the pore, which could mediate the opening and closure of the channel, in line with several previous studies (Figures 7C,D) (Sarkar and Saha, 2020; Monje-Galvan and Voth, 2021). Both models remain in the closed conformation, and the transition from the closed to the open state is not observed throughout the simulations. While the channel is not well-hydrated in the refined model, the hydration profile of the homology model seems to indicate a well-hydrated channel. However, as discussed above, the hydration of the homology model is not due to water molecules passing through the channel but rather being trapped inside the membrane (Figures 7E,F).

### Repurposing drugs targeting the E protein through virtual screening

**FIGURE 8:**
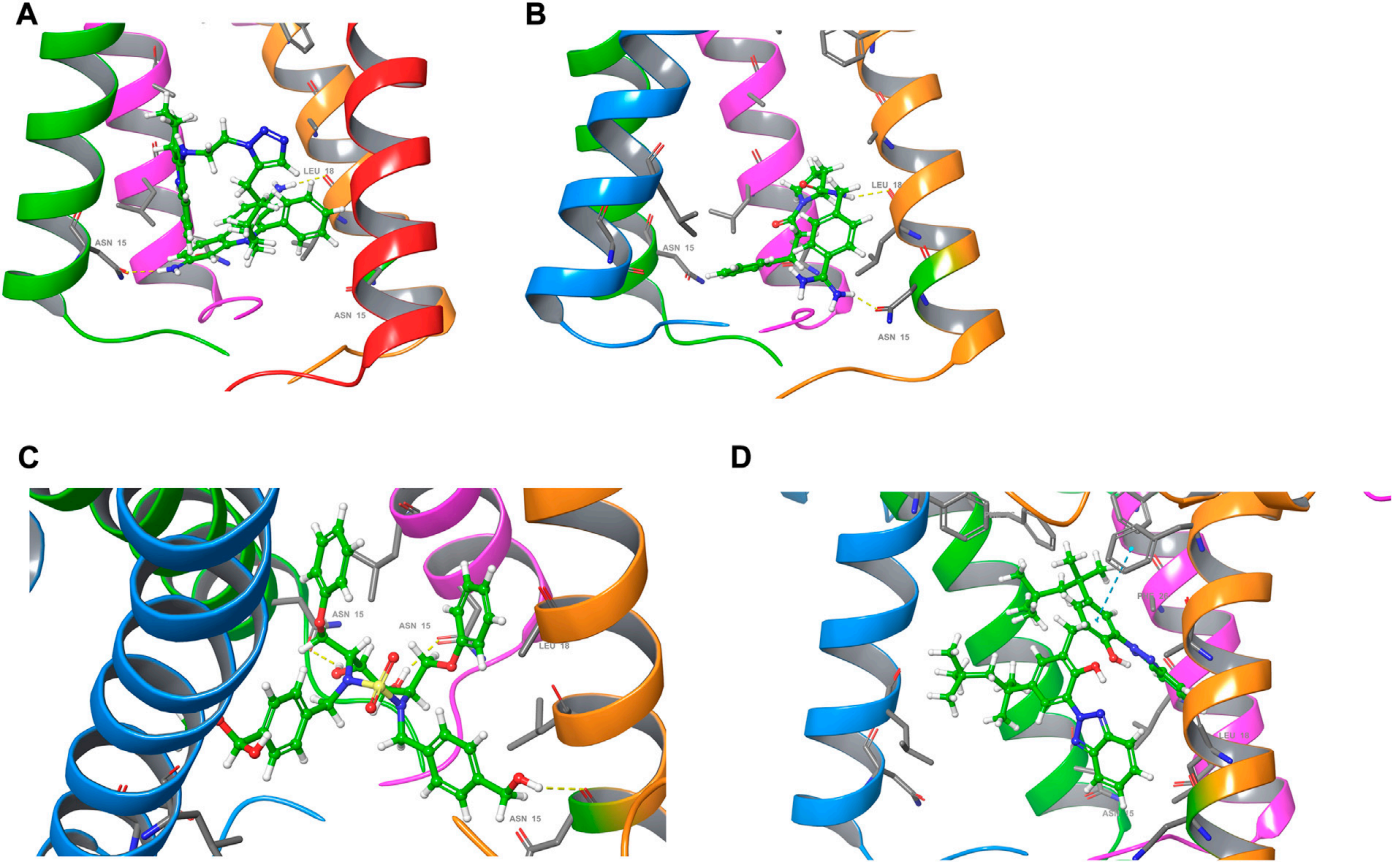
Docking poses of **(A)** DB03005, **(B)** DB06942, **(C)** DB04042 and **(D)** DB11262. For clarity, one of the chains of the E protein is not shown.

The ion channel formed by the pentameric E protein is critical to the viral assembly and maturation (Verdiá-Báguena et al., 2012; Nieto-Torres et al., 2015; Cabrera-Garcia et al., 2021). Impaired ion conductivity caused decreased virulence and reduced viral infection (Boscarino et al., 2008; Castaño-Rodriguez et al., 2018; Xia et al., 2021). Several studies sought to identify the E protein channel blockers by *in vitro* cell-based assays and *in silico* screening (Dey et al., 2020; Das et al., 2021; Mukherjee et al., 2021; Xia et al., 2021). Given a better equilibrated and likely more physiologically relevant structural model of the SARS-CoV-2 E protein, we conducted virtual screening of a library of FDA-approved, investigational and experimental drugs to discover potential E protein ion channel inhibitors. The drug library containing 9,213 compounds was obtained from the DrugBank and screened *in silico* by Schrodinger Glide on the Ohio Supercomputer Center (Ohio-Supercomputer-Center, 1987; Halgren et al., 2004; Wishart et al., 2017). The docking results were ranked by Glide score (gscore) and the top 100 results were visually inspected. The entire channel can be divided by the bottleneck residue PHE26 into an upper and a lower channel. All the docking poses are located in the lower channel. Many of the top-ranking compounds form H bonds with ASN15 side chain at the bottom of the channel. The mutation of ASN15, such as N15A, was found to reduce ion conductance (Verdiá-Báguena et al., 2012). Additionally, some top-ranking compounds form H bonds with the backbone of LEU18. Only one molecule (DB11262) establishes π-π stacking interaction with PHE26. 20 compounds were selected from the top-ranking results based on the key interactions formed with the E protein (Table 1) and their physiochemical properties and mode of action/indications were summarized in Supplementary Tables S2, S3. The representative binding poses of the selected compounds are shown in Figure 8; Supplementary Figure S8. We found that most of these compounds are beyond the rule-of-five scope, suggesting that in order to fully block the channel, large compound size seems necessary. Interestingly, among these twenty compounds, two molecules (DB12138 and DB11871) might also inhibit the SARS-CoV-2 main protease according to the screening work by Sharma et al. (Sharma et al., 2021). These results might provide an alternative starting point for the development of therapeutics against COVID-19.

**TABLE 1:** List of 20 top-ranked compounds and their Glide score (gscore) and the key interactions formed with the E protein.

Database ID	Gscore (kcal/mol)	Molecular weight (g/mol)	cLOGP	Key interactions/H bonds
DB02009	−10.9	652.8	4.1	N15
DB02629	−10.8	688.7	3.2	-
DB12138	−10.6	700.3	6.9	-
DB02704	−10.4	648.8	4.7	-
DB04190	−10.3	636.7	3.6	-
DB11262	−10.3	658.9	11.8	F26
DB03005	−10.3	661.9	3.1	N15, L18
DB06942	−10.0	378.5	1.9	N15
DB06401	−9.9	470.6	6.0	N15, L18
DB05038	−9.8	711.7	4.0	N15
DB11871	−9.8	617.8	3.1	N15, L18
DB04172	−9.7	612.8	2.8	N15
DB06494	−9.7	667.7	6.9	N15
DB14879	−9.7	752.2	-2.9	N15
DB01329	−9.7	645.7	-0.9	N15
DB04042	−9.6	664.8	3.1	N15
DB04421	−9.6	747.5	-8.8	N15
DB03276	−9.5	688.7	2.6	N15
DB15982	−9.5	562.6	5.6	N15
DB03300	−9.3	700.5	-4.3	N15

## Conclusion

The SARS-CoV-2 envelope (E) protein is thought to play essential roles in the viral maturation and pathogenesis, but its structure in physiological conditions is not well understood. This has greatly hampered our fundamental understanding of E protein-mediated membrane fusion and viral assembly. This study began with a homology model of the E protein and investigated its structure and dynamics in a POPC lipid bilayer with molecular dynamic (MD) simulations. Our results show that the homology model, especially the C-terminal domain, is not stable when embedded in the lipid bilayer. The H3 helices of four monomers are found to move to the lipid headgroup region in the simulations. Based on this observation, we built a refined E protein structure by replacing the H3 structure with the one fully equilibrated in the MD simulations. Not surprisingly, the refined model displays smaller structural fluctuations. The stability of the refined model likely arises from the more H bonds and favorable interactions of the H3 helices with both lipids and water.

The presence of the SARS-CoV-2 E protein induces a local membrane curvature - thinning and softening the surrounding lipid bilayer. This could partially facilitate the accommodation of the unstable H3 helices in the original homology model. Furthermore, our simulations indicate that both models represent a closed channel with a hydrophobically occluded pore. Finally, given a better E protein structural model, we conducted virtual screening against a library of 9,213 FDA drugs and discovered 20 compounds that could potentially function as E protein channel blockers.

The refined structural model for the SARS-CoV-2 E protein is offered as a platform to aide in the interpretation of experimental and computational results as the community continues to investigate the role of the E protein viral maturation and pathogenesis. As we demonstrate, it is also expected that this refined model will facilitate the development anti-COVID-19 drugs targeting the E protein.

## Methods

### Homology modeling and system preparation

The primary sequences of SARS-CoV E protein (Accession number: AYV99820.1) and SARS-CoV-2 E protein (Accession number: QII57162.1) were retrieved from NCBI protein database at www.ncbi.nlm.nih.gov/protein/. These two proteins have high similarity (94.74% identity) in sequence, which were compared at http://blast.ncbi.nlm.nih.gov. The structure of SARS-CoV E protein (PDB 5X29) was used as the template structure for homology modeling by Modeller 9.1 (Šali and Blundell, 1993). The sequence of the SARS-CoV-2 E protein was aligned with the structure of the SARS-CoV E protein using the align2d command in MODELLER. 10,000 3D models of the SARS-CoV-2 E protein were generated using the AutoModel class. The results were plotted by their DOPE score and molpdf values. Three structures with the lowest DOPE score and molpdf values (query.B99990502, query.B99990876, and query.B99990918) were visually inspected and query.B99990502 was selected as the final model due to its smallest structural deviation from the template. The model was submitted to SAVES v6.0 developed by the DOE lab at UCLA (https://saves.mbi.ucla.edu/) and Molprobity developed by Williams et al. at Duke University (http://molprobity.biochem.duke.edu/) (Williams et al., 2018) for stereochemical quality check. The final pentameric structure of SARS-CoV-2 E protein was built with the homology modeling structure by GalaxyHomomer (https://galaxy.seoklab.org/cgi-bin/submit.cgi?type=HOMOMER). Each pentameric E protein structure was embedded in a membrane bilayer containing 150 1-palmitoyl-2-oleoyl-glycero-3-phosphocholine (POPC) molecules on each leaflet using CHARMM-GUI Membrane Builder (Jo et al., 2008; Wu et al., 2014). The bury depth was determined by PPM (one of the orientation options on CHARMM-GUI), which predicts the membrane embedding orientation and depth for a given input protein structure at https://opm.phar.umich.edu/ppm_server2_cgopm. Approximately 20,000 TIP3P water molecules and 0.15 M NaCl were added to each system to ensure electric neutrality.

### AA-MD simulations

All simulations were performed using AMBER following the 6-step protocol provided on the CHARMM-GUI web-server (D.A. Case, 2018). The Lipid14 force field was used for lipids (Dickson et al., 2014). The ff14SB was used for the protein (Maier et al., 2015). The TIP3P was used for water molecules (Price and Brooks, 2004). The AMBER-99 force field was used for ions (Chen and Pappu, 2007). The non-bonded pair list was updated every 1,000 steps with the cutoff of 12 Å. Long-range electrostatic interactions were calculated using particle mesh Ewald (PME) method (Darden et al., 1993). Bonds involving hydrogen were constrained with the SHAKE algorithm (Ryckaert et al., 1977). The temperature was maintained at 303.15 K with a friction coefficient of 1.0 ps^−1^ using the Langevin dynamics algorithm and the pressure was maintained at 1.0 bar using the Berendsen pressure coupling algorithm (Berendsen et al., 1984). The pressure was coupled semi-isotropically, in which the *x* and *y* directions were coupled together, and the *z* direction fluctuates independently. Three independent 2 μs simulations were run for the homology and the refined models, respectively. One simulation of the homology model was run at 323.15 K for 1 μs. One simulation of a pure lipid bilayer consisting of 300 POPC molecules (150 on each leaflet) was run at 303.15K for 200 ns.

### Virtual screening

A compound library containing 9,213 FDA-approved, investigational and experimental drugs was obtained from the DrugBank (Wishart et al., 2017). Each of these compounds was docked into the E protein channel by Glide XP (Madhavi Sastry et al., 2013) from the Schrodinger suite installed on the Ohio Supercomputer Center (Ohio-Supercomputer-Center, 1987). Ligand structures were prepared by Ligprep (Schrödinger, 2021a). The protein structure was prepared by Protein Preparation Wizard (Madhavi Sastry et al., 2013). The docking results were analyzed based on docking scores and visual inspection. The images of the docking poses were rendered by Maestro (Schrödinger, 2021b).

### Analysis

All the images, structures and trajectories were visualized and rendered on the Visual Molecular Dynamics (VMD) software package (Humphrey et al., 1996) and Pymol (Schrodinger, 2015) except Figures 7A,B which were generated by The Channel Annotation Package (Klesse et al., 2019). All figures were plotted with OriginPro (OriginLab Corporation, Northampton, MA, United States). All analyses were performed with CPPTRAJ or Python using in-house scripts (Roe and Cheatham, 2013).

Membrane thickness is calculated using the positions of phosphate atoms of the lipid head. The upper and lower surface are represented by the two-dimensional Fourier series to the order of 3 in both *x* and *y* direction. Coefficients are determined by fitting the series to the phosphate atoms positions for every frame (the first 500ns are removed).

$$z=a_{0}+\sum_{m=1}^{3}\sum_{n=1}^{3}a_{mn}\sin\left(\frac{n\pi x}{a}\right)\sin\left(\frac{m\pi y}{b}\right),0<x^{\prime }<a,\;0<y^{\prime }<b$$ (1)

For each phosphate atom, the thickness at that position is calculated by $z_{upper}-z_{lower}$ .

The order parameter is defined as:

$$S_{CH}=-\frac{<3\cos^{2}\alpha -1>}{2}$$ (2)

where α is the angle between the C-H bond and the bilayer normal.

Tilt is defined as the angle between the bilayer normal and a vector that connects the COM of the phosphate and glyceride C2 atom with the COM of the three terminal carbon atoms on the lipid tail of POPC (Khelashvili et al., 2013). The splay angle of a lipid is the average angle of its head-to-tail vector with that of surrounding lipids within 10 Å. The S_Tilt_ and S_Splay_ are derived analogously from Eq. 2 for the tilt and splay angles, respectively.

## Data Availability

The raw data supporting the conclusions of this article will be made available by the authors, without undue reservation. The homology model of the E protein, the refined model of the E protein, and computational models of the E protein bound with drug molecules are available at https://u.osu.edu/chenglab/research/.
