# Supplementary material for: Refinement of SARS-CoV-2 envelope protein structure in a native-like environment by molecular dynamics simulations
Source: Front Mol Biosci. 2022 Oct 10;9:1027223. doi: 10.3389/fmolb.2022.1027223 (PMC9589232; doi:10.3389/fmolb.2022.1027223)
Supplement: Supplementary file 1 [file DataSheet1.docx]

Supplementary Material

# Supplementary Figures and Tables

## Supplementary Figures

**A**
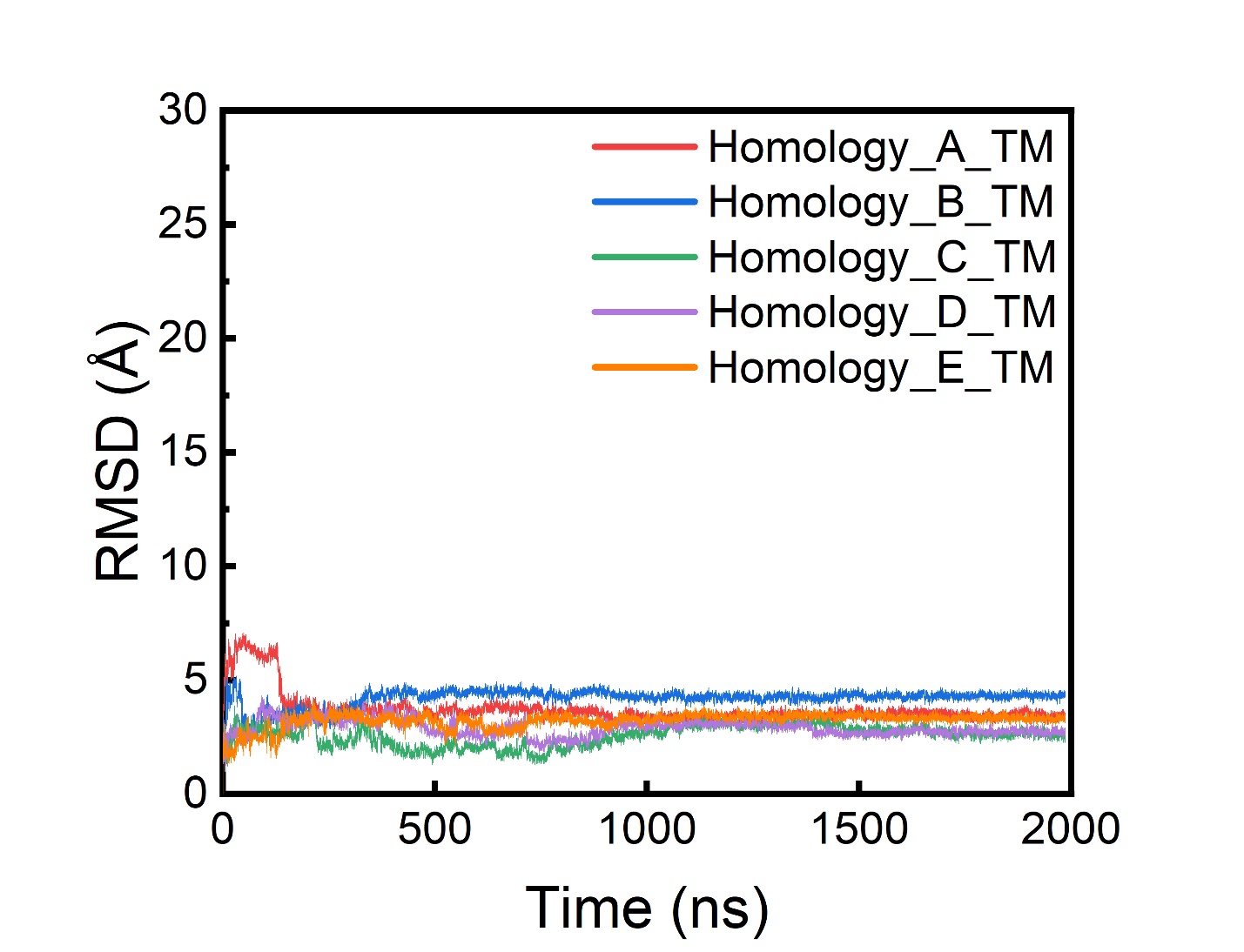
**B**
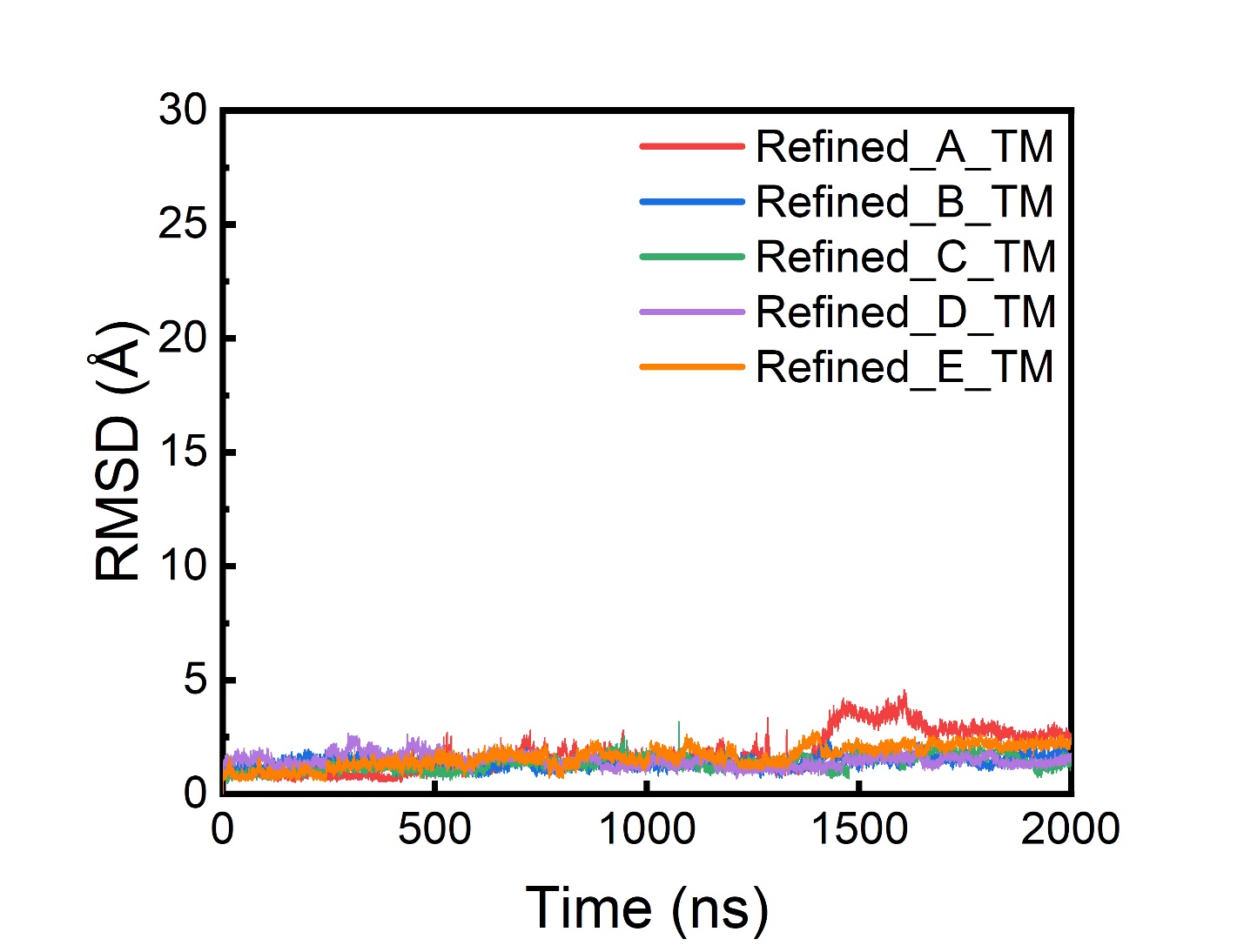
**C**
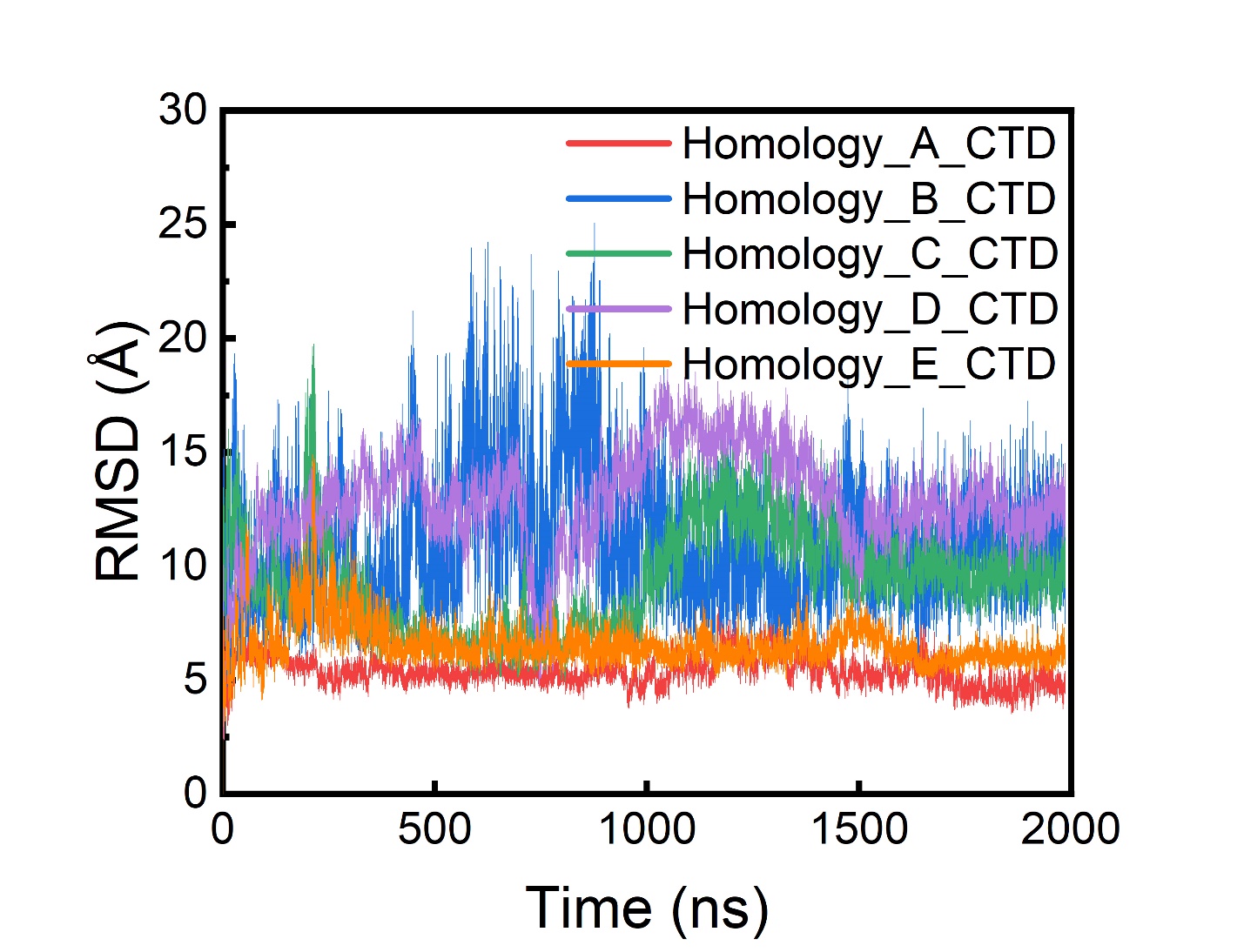
**D**
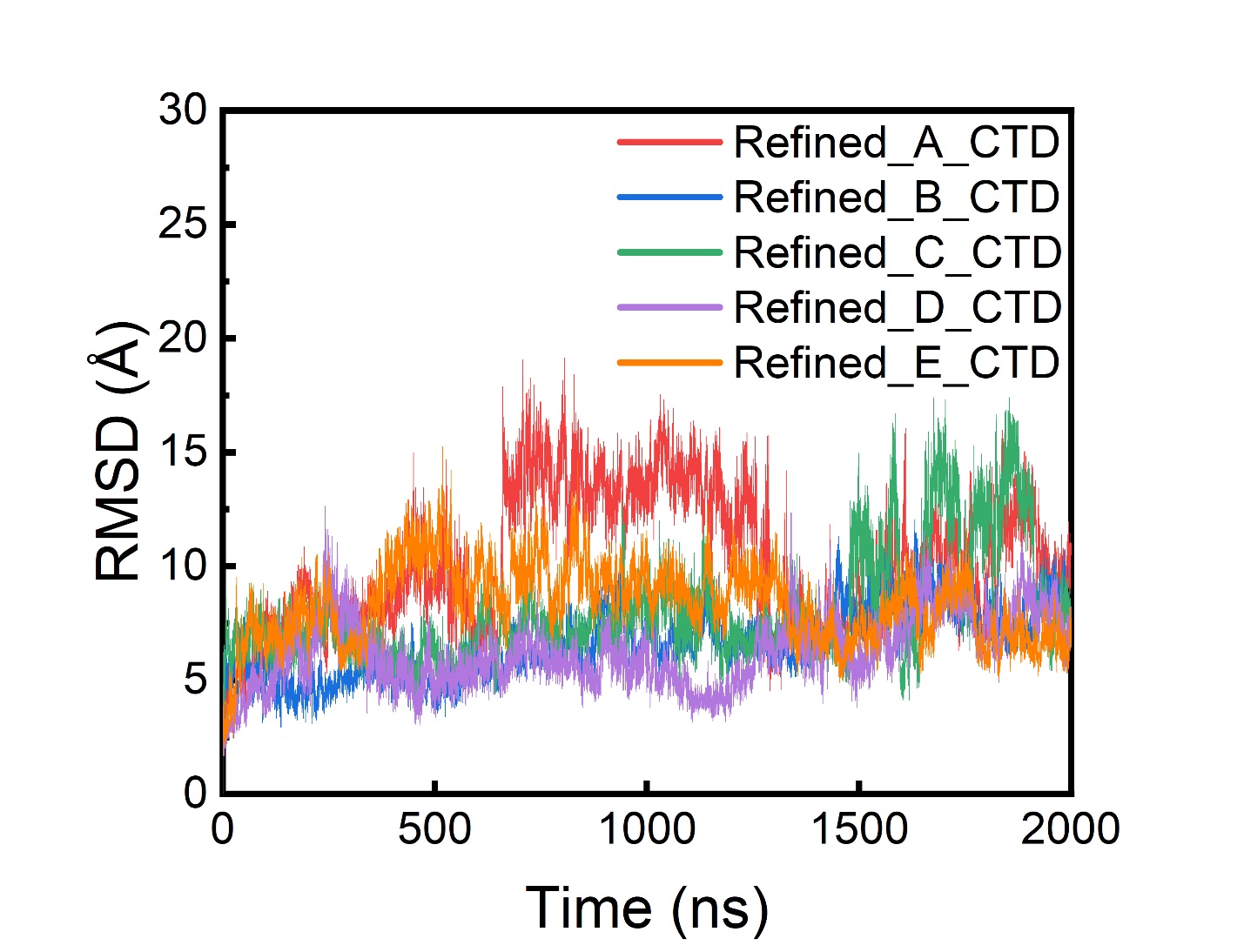


**Figure S1.** RMSDs of TM regions of **(A)** the homology and **(B)** the refined model. RMSDs of CTDs of **(C)** the homology and **(D)** the refined model.

**A**
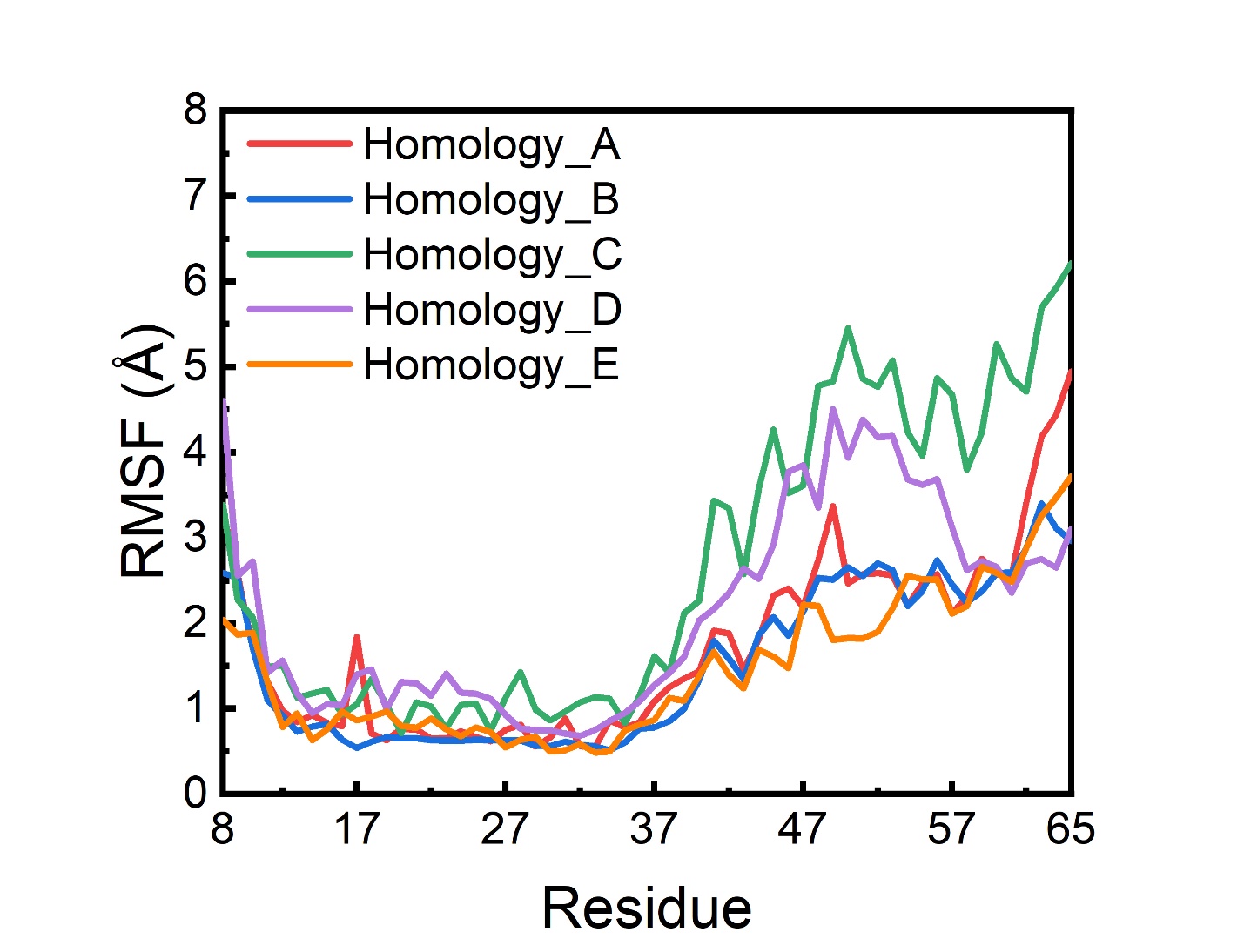
**B**
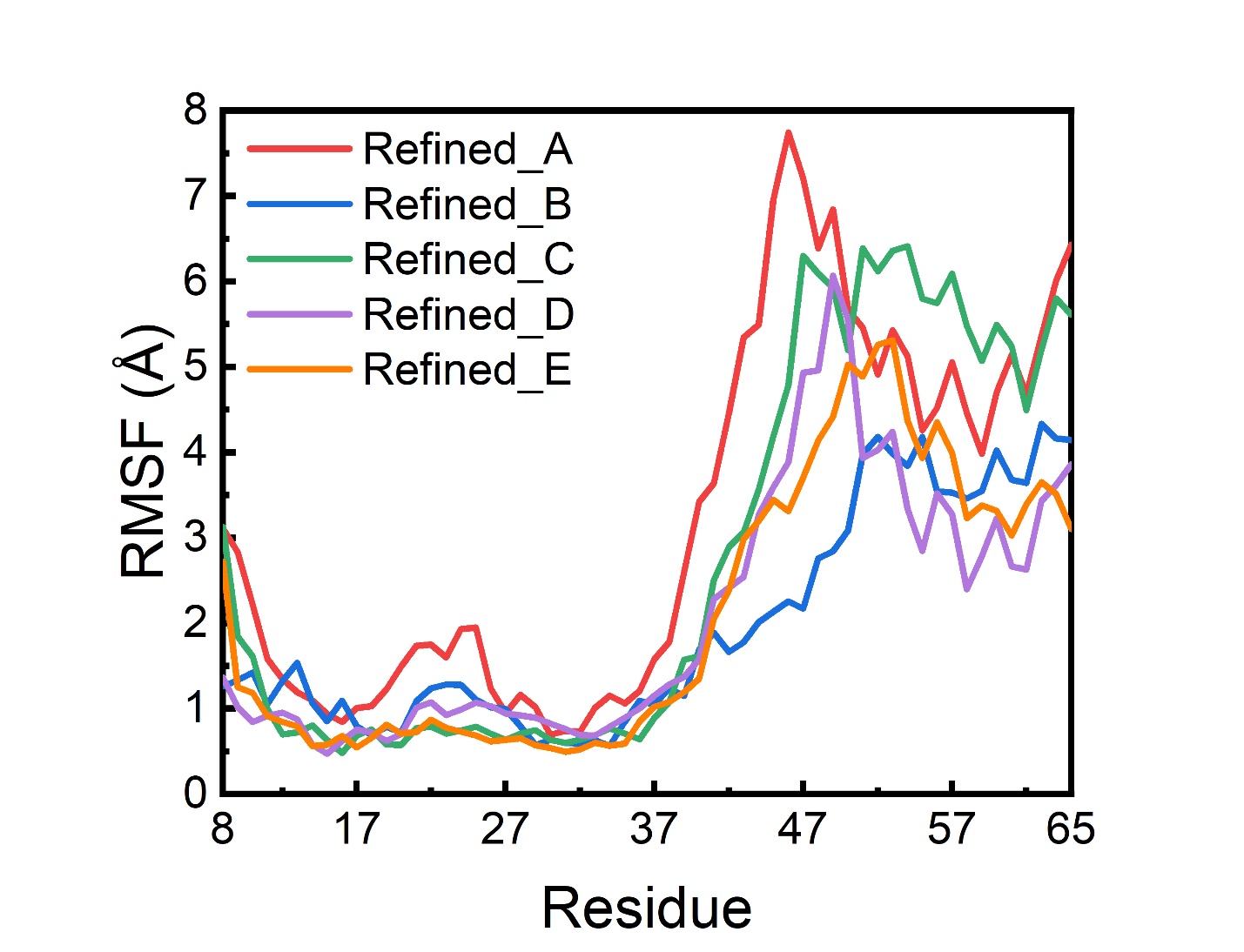


**Figure S2.** RMSF of **(A)** the homology model and **(B)** the refined model.

**A**
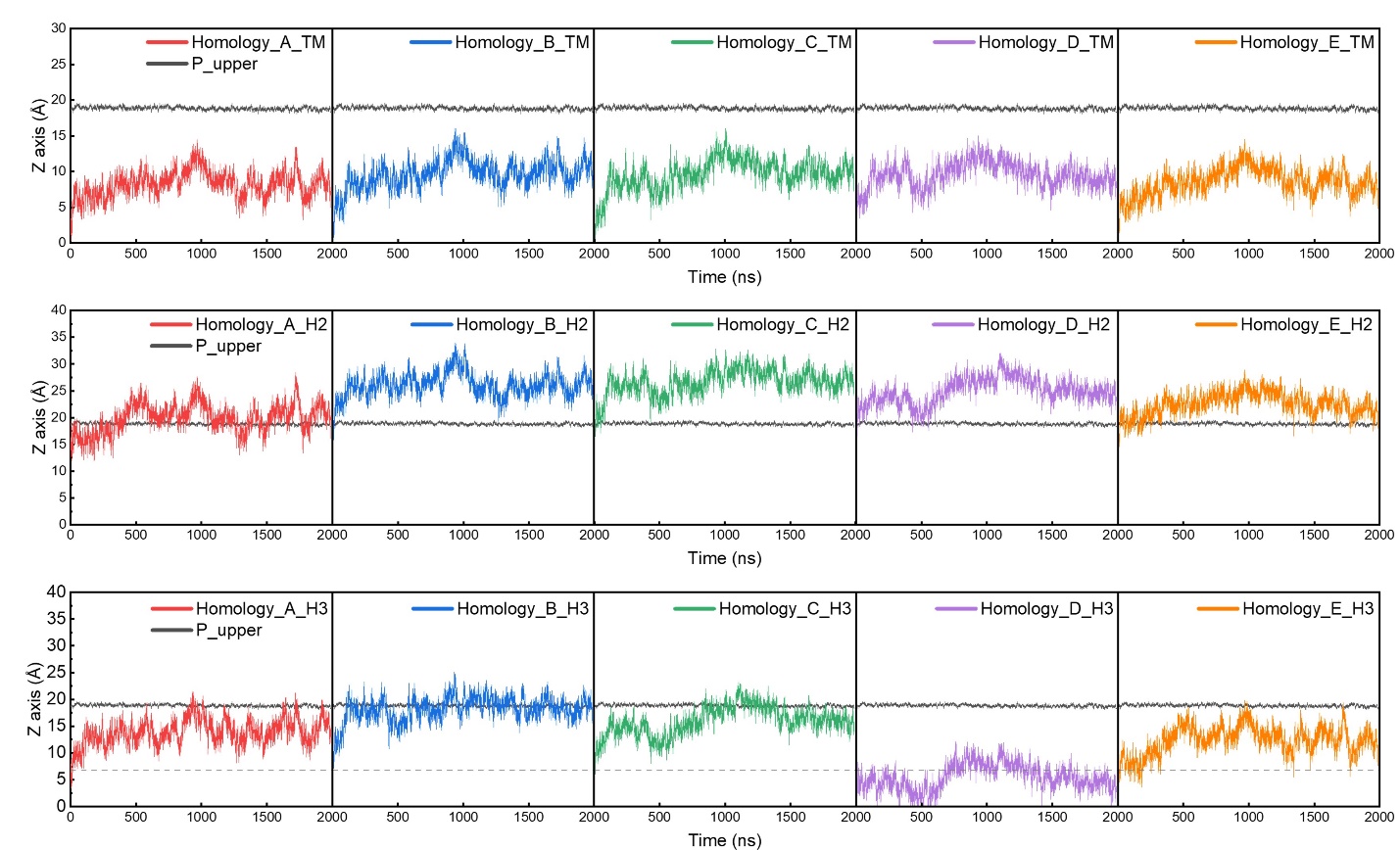


**B**
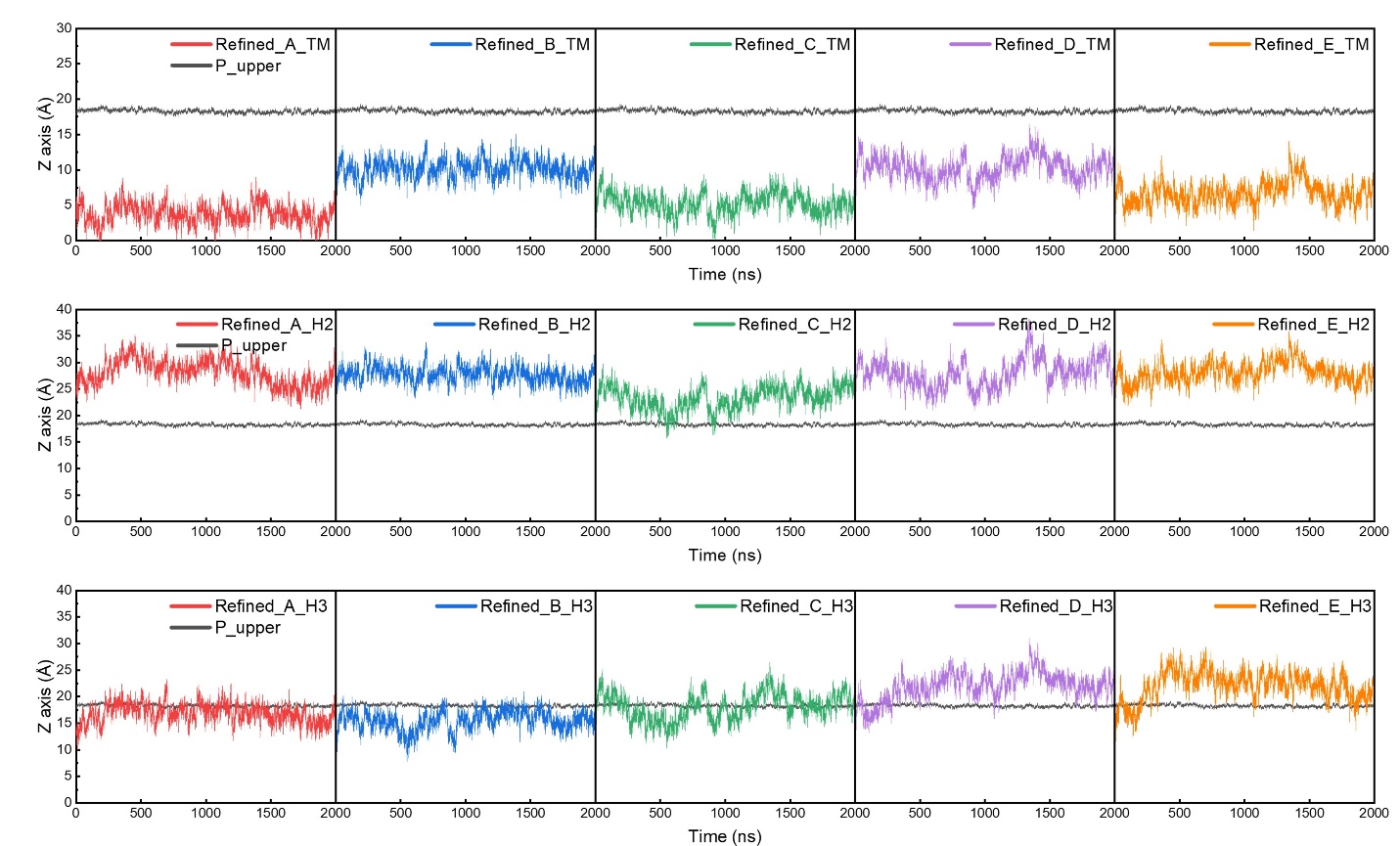


**Figure S3.** COM movement of TM regions (upper), H2s (middle), H3s (lower) and the phosphorus atoms on the upper leaflet along the membrane normal in the simulations of **(A)** the homology model and **(B)** the refined model. The initial position of H3’s COM in the homology model is shown as a dashed line for reference.

**
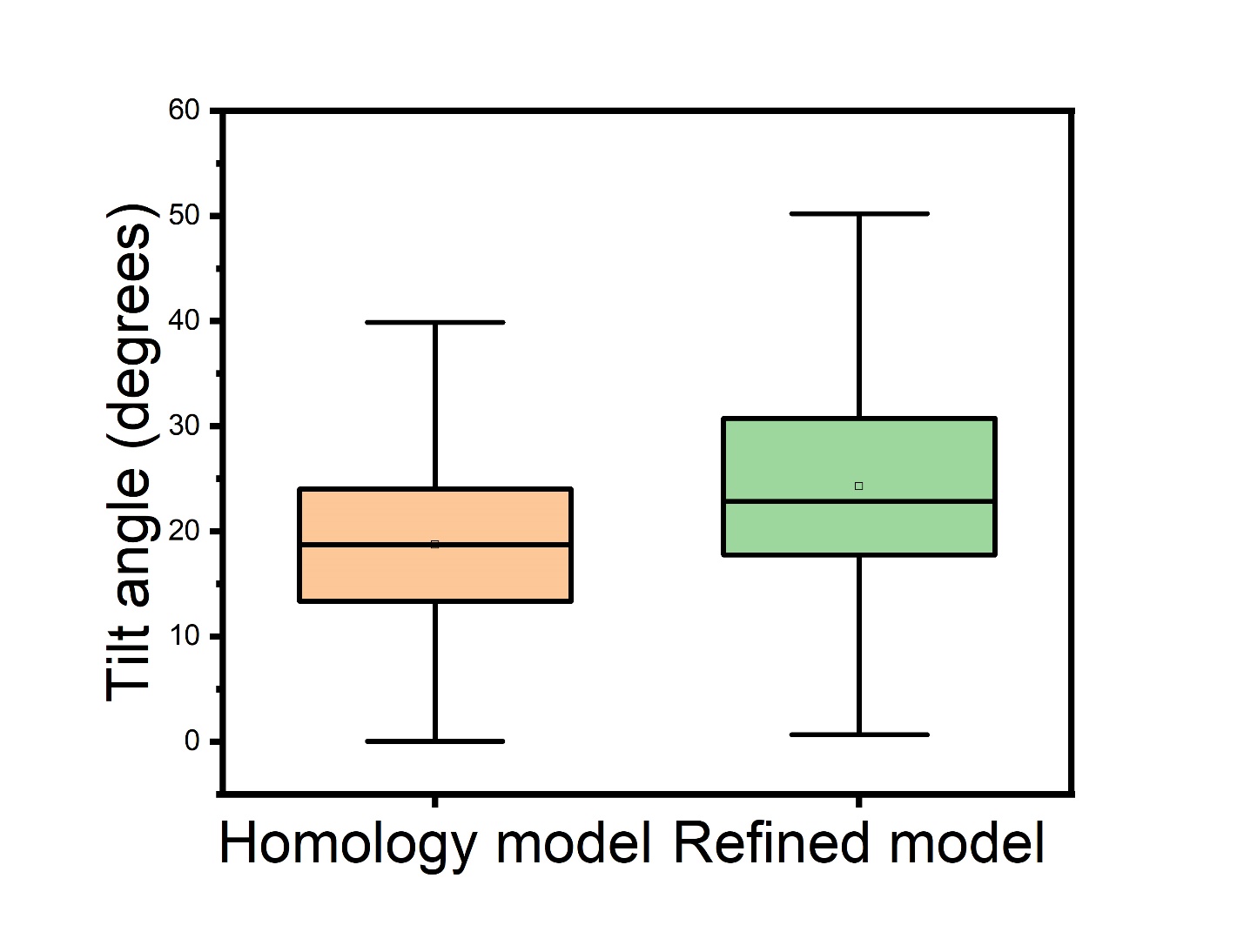
Figure S4.** The distribution of the tilt angle of the TM regions in both models.


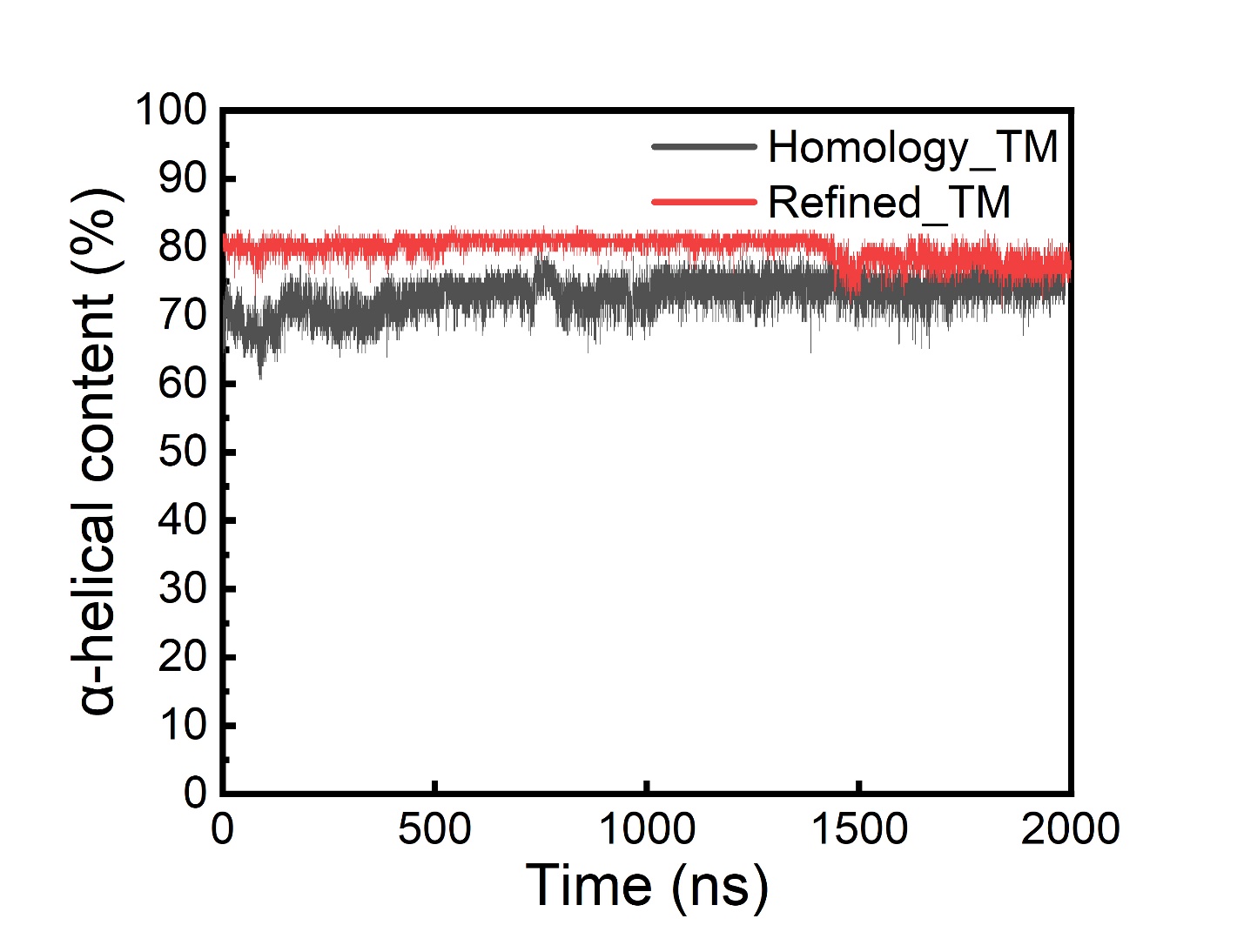


**Figure S5.** Time-evolution of the alpha helical content of the TM regions in both models.

**A**
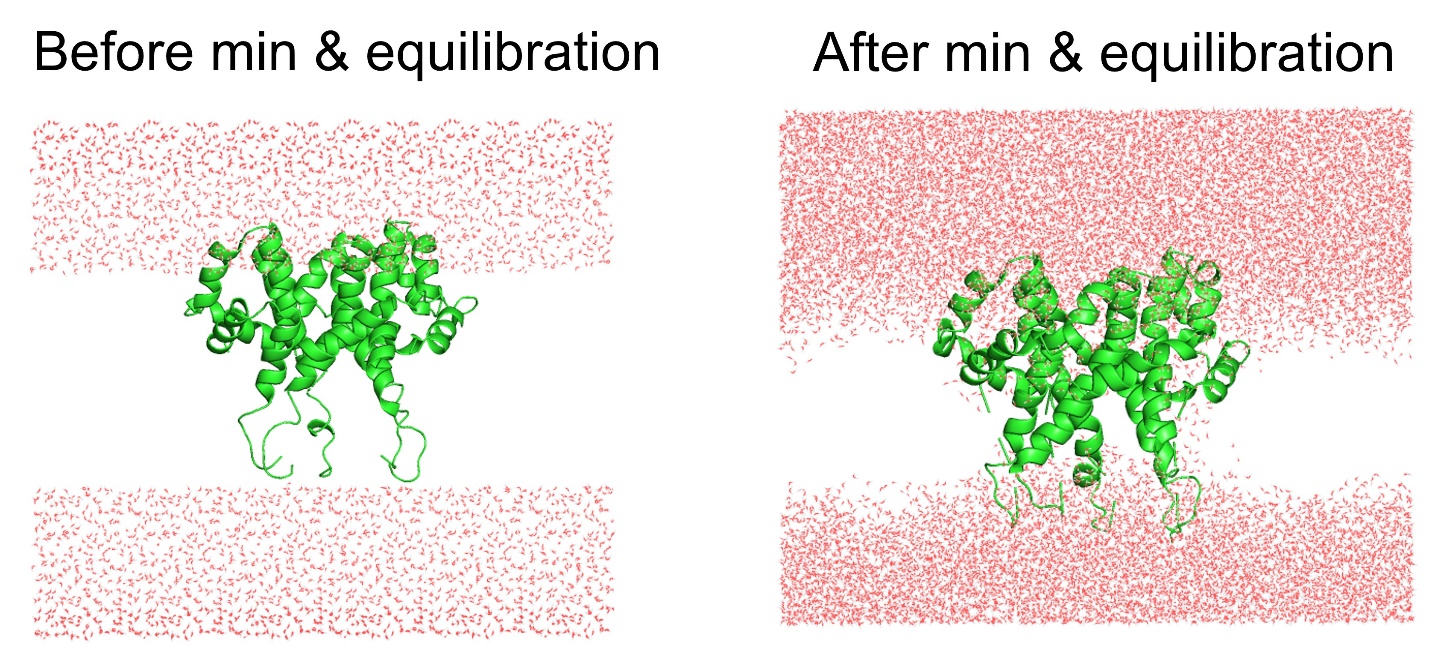
**B**
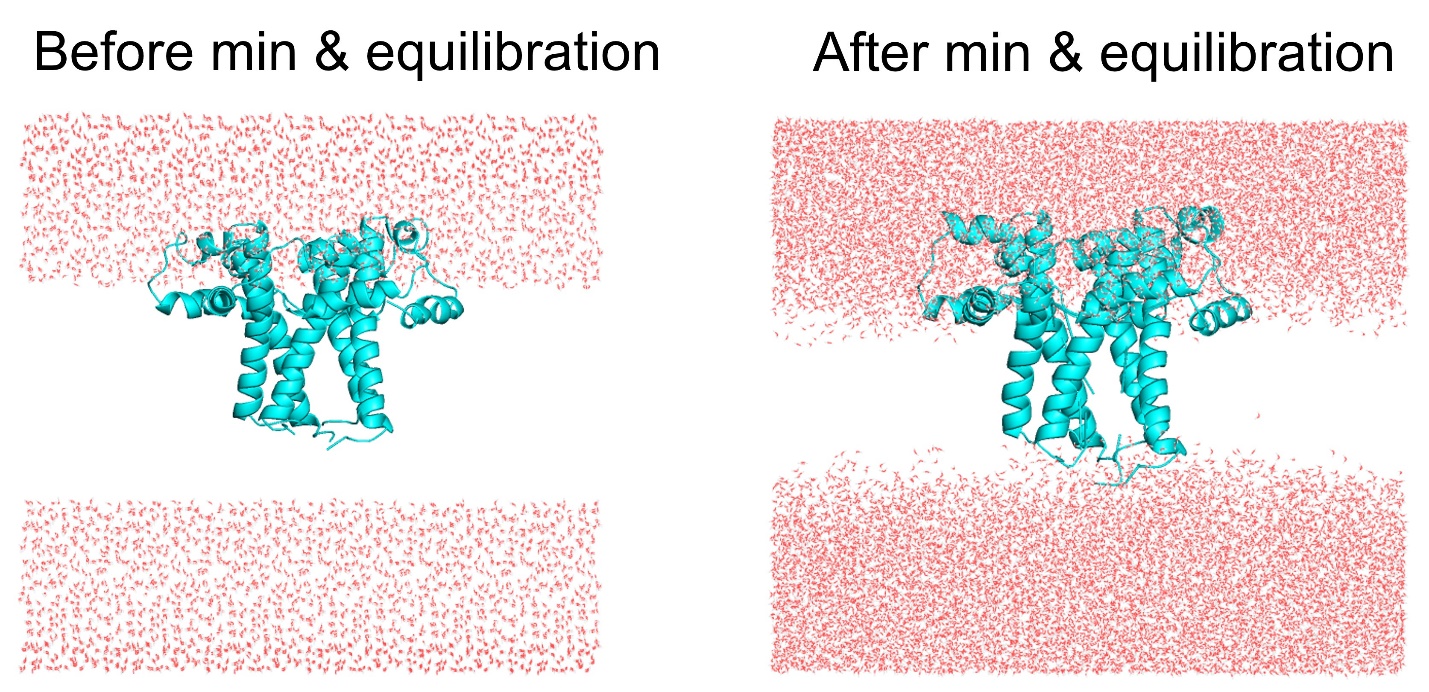


**Figure S6.** The structure of **(A)** the homology model and **(B)** the refined model system before and after energy minimization and equilibration. The lipids and ions were removed for clarity and only protein and water molecules were displayed.


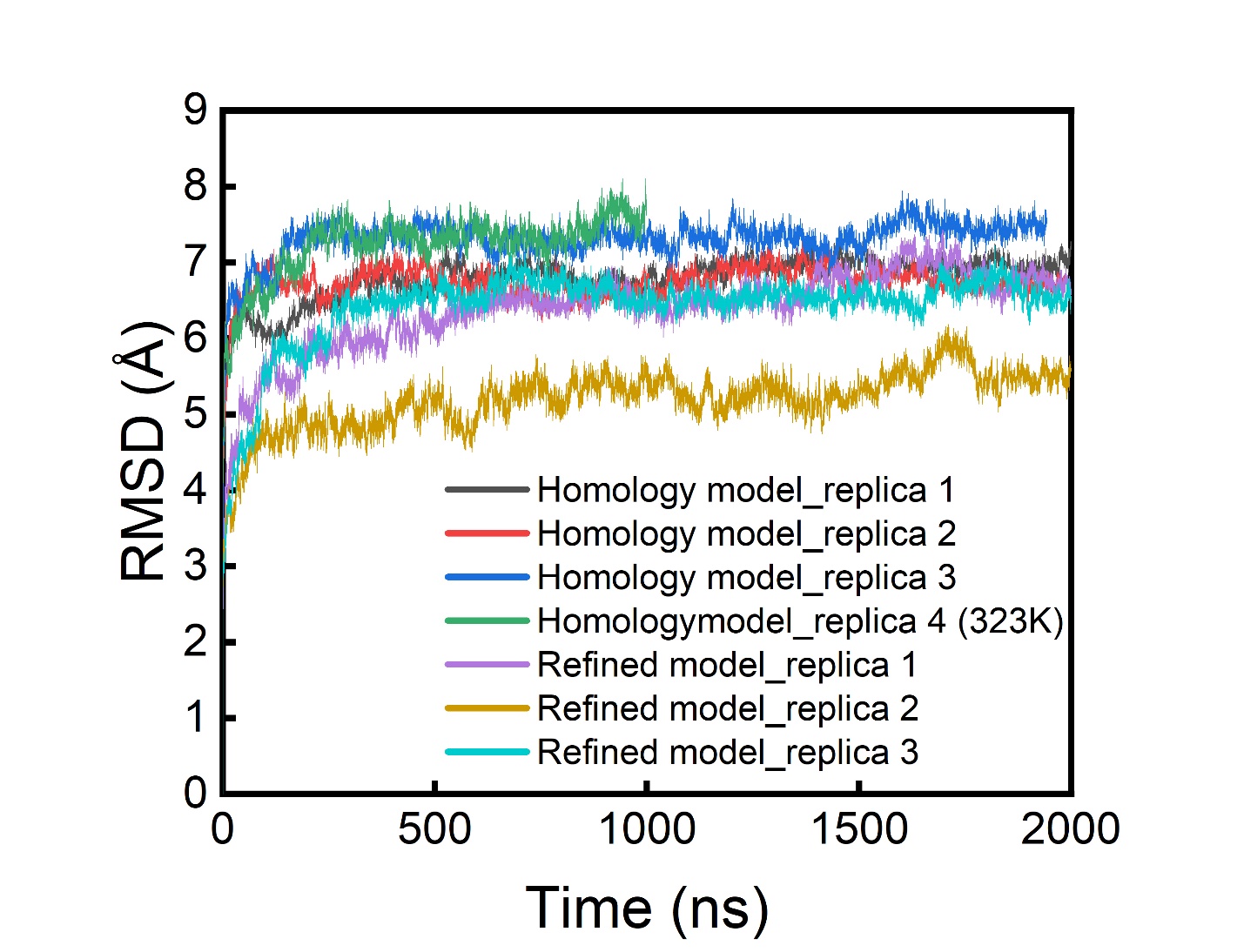


**Figure S7.** The RMSD of all AA-MD trajectories.


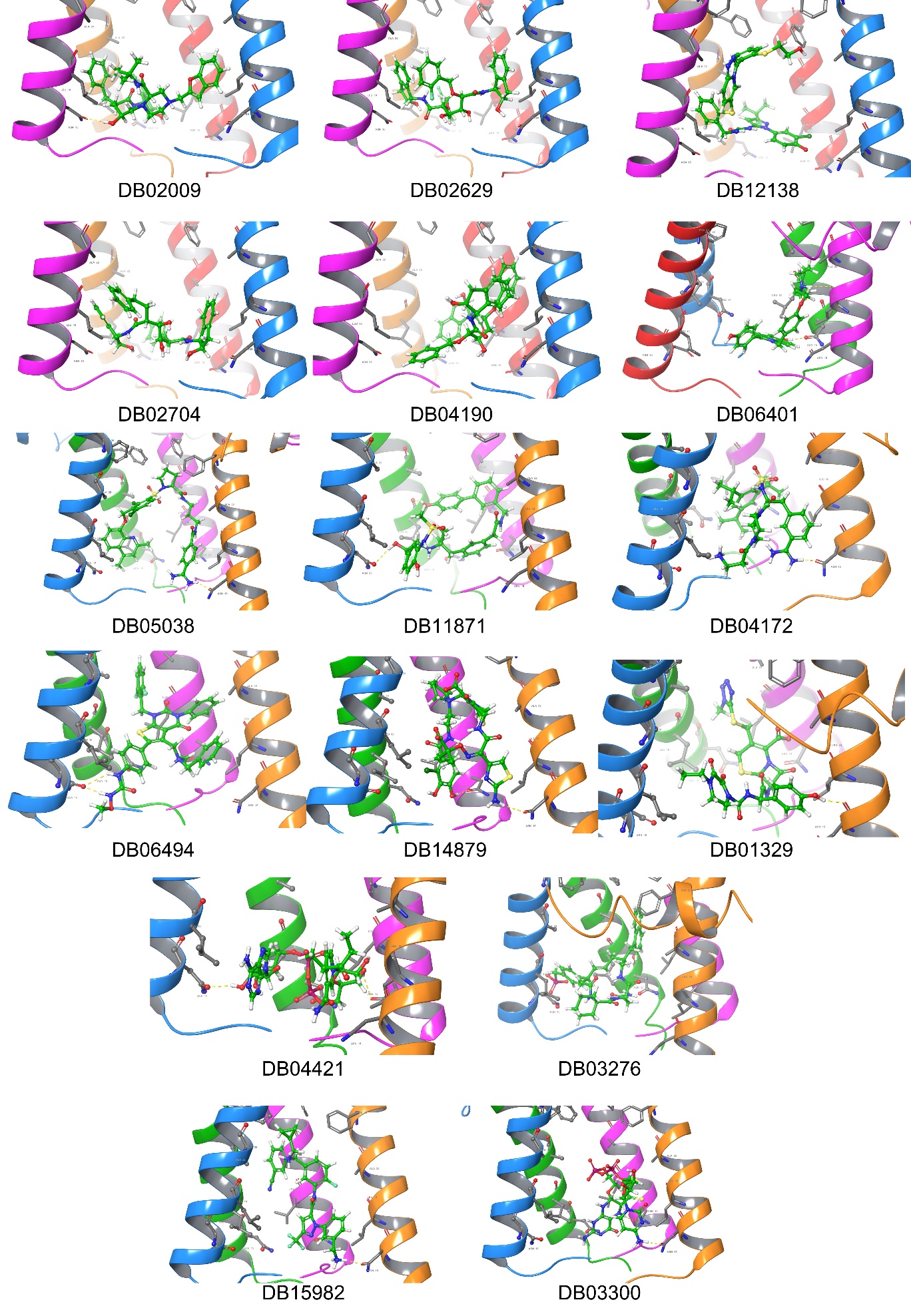


**Figure S8.** The binding poses of the selected compounds from virtual screening.

## Supplementary Tables

**Table S1.** The structural qualities of the experimentally determined and computationally predicted structures.

| **Structure** | **Ramachandran plot summary^a^** | **ERRAT Overall Quality Factor** | **Clash score^b^** |
| --- | --- | --- | --- |
| Homology model (res 8-65) | 92.6%/5.6%/1.9%/0.0% | 88 | 3.01 |
| 5X29 (res 8-65) | 83.3%/16.7%/ 0.0%/0.0% | 36.7 | 8.29 |
| 7K3G (res 8-38) | 96.4%/0.0%/0.0%/3.6% | 47.8 | 12.25 |
| Feig model (res 1-75) | 95.7%/1.4%/1.4%/1.4% | 100 | 0.82 |
| Swiss model (res 8-65) | 85.2%/13.0%/1.9%/0.0% | 61.2 | 1.27 |
| I-TASSER model (res 1-75) | 87.1%/12.9%/0.0%/0.0% | 92.5 | 0 |

1. Residues in most favored/additional allowed/generally allowed/disallowed regions.
2. Clash score is the number of atoms having serious steric overlaps (> 0.4 Å) per 1000 atoms.

**Table S2.** List of the 20 selected compounds and their physiochemical properties.

| **DATABASE ID** | **ACCEPTOR COUNT** | **DONOR COUNT** | **POLAR SURFACE AREA** |
| --- | --- | --- | --- |
| DB02009 | 7 | 4 | 118.3 |
| DB02629 | 10 | 6 | 157.6 |
| DB12138 | 8 | 4 | 129.6 |
| DB02704 | 6 | 6 | 139.1 |
| DB04190 | 7 | 5 | 137.4 |
| DB11262 | 6 | 2 | 101.9 |
| DB03005 | 6 | 3 | 111.6 |
| DB06942 | 3 | 3 | 99.3 |
| DB06401 | 4 | 2 | 57.9 |
| DB05038 | 8 | 4 | 167.6 |
| DB11871 | 8 | 6 | 148.0 |
| DB04172 | 7 | 4 | 162.7 |
| DB06494 | 8 | 2 | 94.2 |
| DB14879 | 15 | 6 | 256.9 |
| DB01329 | 13 | 4 | 220.3 |
| DB04042 | 11 | 4 | 149.2 |
| DB04421 | 19 | 7 | 338.2 |
| DB03276 | 7 | 6 | 187.9 |
| DB15982 | 9 | 3 | 108.8 |
| DB03300 | 18 | 11 | 311.4 |

**Table S3.** List of the 20 selected compounds and their mode of action.

| **DATABASE ID** | **DRUG NAME** | **Drug Groups** | **Mode of Action** | **Indications** |
| --- | --- | --- | --- | --- |
| DB02009 | L-756423 | Experimental | HIV-1 protease inhibitor | NA |
| DB02629 | N,N-[2,5-O-di-2-fluoro-benzyl-glucaryl]-di-[1-amino-indan-2-ol] | Experimental | HIV-1 protease inhibitor | NA |
| DB12138 | PF-03715455 | Investigational | p38 MAPK inhibitor | Chronic Obstructive Pulmonary Disease (COPD), Asthma |
| DB02704 | (2R,3R,4R,5R)-3,4-Dihydroxy-N,N'-bis[(1S,2R)-2-hydroxy-2,3-dihydro-1H-inden-1-yl]-2,5-bis(2-phenylethyl)hexanediamide | Experimental | HIV-1 protease inhibitor | NA |
| DB04190 | 2,5-dibenzyloxy-3-hydroxy-hexanedioic acid bis-[(2-hydroxy-indan-1-yl)-amide] | Experimental | HIV-1 protease inhibitor | NA |
| DB11262 | Bisoctrizole | Approved | Absorbing UV rays | Sunscreens |
| DB03005 | 3,8-Diamino-6-Phenyl-5-[6-[1-[2-[(1,2,3,4-Tetrahydro-9-Acridinyl)Amino]Ethyl]-1h-1,2,3-Triazol-5-Yl]Hexyl]-Phenanthridinium | Experimental | Acetylcholinesterase inhibitor | NA |
| DB06942 | N-(4-carbamimidoylbenzyl)-1-(3-phenylpropanoyl)-L-prolinamide | Experimental | Thrombin Inhibitor | NA |
| DB06401 | Bazedoxifene | Approved | Selective estrogen receptor modulator | Prevention of postmenopausal osteoporosis |
| DB05038 | Anatibant | Investigational | Bradykinin B2 receptor antagonist | Traumatic brain injury |
| DB11871 | Benzeneacetamide | Investigational | β2 adrenoreceptor agonist | Chronic Obstructive Pulmonary Disease (COPD), Asthma |
| DB04172 | [2,4,6-Triisopropyl-Phenylsulfonyl-L-[3-Amidino-Phenylalanine]]-Piperazine-N'-Beta-Alanine | Experimental | Urokinase-type plasminogen activator | NA |
| DB06494 | Sufugolix | Investigational | Selective antagonist of the gonadotropin-releasing hormone receptor | Endometriosis and uterine leiomyoma |
| DB14879 | Cefiderocol | Approved | Penicillin-binding proteins inhibitor | Multi-drug-resistant Gram-negative bacteria |
| DB01329 | Cefoperazone | Approved | Bacterial cell wall synthesis inhibitor | Pseudomonas bacterial infections |
| DB04042 | 2-[4-(Hydroxy-Methoxy-Methyl)-Benzyl]-7-(4-Hydroxymethyl-Benzyl)-1,1-Dioxo-3,6-Bis-Phenoxymethyl-1lambda6-[1,2,7]Thiadiazepane-4,5-Diol | Experimental | HIV-1 protease inhibitor | NA |
| DB04421 | Nicotinamide adenine dinucleotide 3-pentanone adduct | Experimental | NA | NA |
| DB03276 | 4-[(10s,14s,18s)-18-(2-Amino-2-Oxoethyl)-14-(1-Naphthylmethyl)-8,17,20-Trioxo-7,16,19-Triazaspiro[5.14]Icos-11-En-10-Yl]Benzylphosphonic Acid | Experimental | Growth factor receptor-bound protein 2 SH2 domain inhibitor | NA |
| DB15982 | Berotralstat | Approved | Selective inhibitor of plasma kallikrein | Prophylaxis of attacks of hereditary angioedema |
| DB03300 | Pterin Cytosine Dinucleotide | Experimental | NA | NA |
